# An unsupervised machine learning approach to evaluate sports facilities condition in primary school

**DOI:** 10.1371/journal.pone.0267009

**Published:** 2022-04-20

**Authors:** Jun Xia, Jihong Wang, Hua Chen, Jie Zhuang, Zhenbo Cao, Peijie Chen

**Affiliations:** 1 School of Kinesiology, Shanghai University of Sport, Shanghai, China; 2 Institute of Urban Development and Management, Tongji University, Shanghai, China; Hefei University of Technology, CHINA

## Abstract

Sports facilities have been acknowledged as one of the crucial environmental factors for children’s physical education, physical fitness, and participation in physical activity. Finding a solution for the effective and objective evaluation of the condition of sports facilities in schools (SSFs) with the responding quantitative magnitude is an uncertain task. This paper describes the utilization of an unsupervised machine learning method to objectively evaluate the condition of sports facilities in primary school (PSSFC). The statistical data of 845 samples with nine PSSFC indicators (indoor and outdoor included) were collected from the Sixth National Sports Facility Census in mainland China (NSFC), an official nationwide quinquennial census. The Fuzzy C-means (FCM) algorithm was applied to cluster the samples in accordance with the similarity of PSSFC. The clustered data were visualized by using *t*-stochastic neighbor embedding (*t*-SNE). The statistics results showed that the application of *t*-SNE and FCM led to the acceptable performance of clustering SSFs data into three types with differences in PSSFC. The effects of school category, location factors, and the interaction on PSSFC were analyzed by two-way analysis of covariance, which indicated that regional PSSFC has geographical and typological characteristics: schools in the suburbs are superior to those in the inner city, schools with more grades of students are configured with better variety and larger size of sports facilities. In conclusion, we have developed a combinatorial machine learning clustering approach that is suitable for objective evaluation on PSSFC and indicates its characteristics.

## Introduction

The prosperity of a country, to some extent, relies on the comprehensive literacy of the young generation, among which physical fitness (PF) is regarded as one of the fundamental and vital elements [1]. Sufficiency of physical activity (PA) at a moderate to vigorous level, recommended by WHO [2], is a well-researched contribution to facilitate health-related PF and reduction of childhood obesity, non-communicable chronic diseases, heart disease, and diabetes [3–7], In general, schoolchildren’s PF and PA are diversified not only by individual characteristics [8] but also by environmental factors [9–11]. Among the latter, in contrast to the family and community, school is widely perceived as the subject of admission and responsibility for the promotion and intervention of schoolchildren’s PA [12]. Previous investigators have identified the positive correlation between the diversity of SSFs and children’s PA [13,14]. Moreover, as the solid foundation of high-quality physical education, the excellence of SSFs motivates schoolchildren to be physically active during recess and lunchtime [15]. The discrepancies in SSFs determine the variance of schoolchildren’s accessibility and availability to school-based PA participation. Therefore, the condition of SSFs can be deemed as the indirect and non-ignorable factor of PA promotion in childhood.

In recent years, several researchers have sought to classify SSFs for the quantification of correlation between differentiated SSFs and students’ PA. Fórnias et al. [16] introduced the “number of PA facilities” variable into the analysis of the relationship between adolescent PA and extracurricular sports activities. This variable was composed of the quantity of six types of SSFs. Lo et al. [17] conducted a cross-sectional study on the Taiwanese adolescent school sports condition and relevant activities in recess time, with the consideration of the investigated schools with or without a school sports field and gymnasium. Haug et al. [18] generated an outdoor facility index (summarized and standardized score of outdoor SSFs numbers) based on a school-level questionnaire for Norwegian primary and secondary schools. The results revealed its correlation with students’ physical activity. Bevans et al. [19] proposed a regression model to evaluate PE effectiveness by interviewing teachers at three school districts in the United States. In this model, school facility resources were rated into three levels, according to teachers’ satisfaction with six types of sports facilities. In essence, existing studies have involved the assessment or classification based on the actual quantity and subjective satisfaction of SSFs. However, few writers have been able to draw on any systematic research into the size factor of SSFs. Meanwhile, extensive researches have been carried out in both developed and developing districts, but the issue in mainland China has rarely been documented. As far as our knowledge, there have been few studies carried out to exert an objective SSFs evaluation for primary schools with high effectiveness and efficiency.

To address the drawback of traditional subjective approaches (users’ comments, questionnaire, interview, grading scales, etc.), the major purpose of this study is aiming to provide a novel combined machine learning method on the feature extraction and evaluation of PSSFC based on the objective SSFs data. Firstly, FCM was utilized to cluster investigated samples with the sample into the same categories. The characteristics of categories in PSSFC are exhibited accordingly, which gives a good understanding of the PSSFC differences within a specific region. Secondly, *t*-SNE was used to perform the visualization of clustered results in the lower dimension to give an explicit illustration of the distribution. Thirdly, a web-crawling method is used to acquire the individual’s location as the supplementary attribution for further analysis. Based on the above results, the two-way ANOVA analysis was conducted to indicate the impact and interaction of the individual’s location and school category on its PSSFC in the same clustered PSSFC type. The results of the experiment showed that the machine learning approach by FCM with *t*-SNE in this paper performs with effective and interpretable performance on the aspect of feature extraction and evaluation and on PSSFC.

## Methodology

### Data collection

This study was based on the Sixth National Sports Facility Census (NSFC, 2014) conducted by the General Administration of Sport of China to survey the quantity, distribution, and purpose of the existing sports facilities in mainland China. The School of Kinesiology at Shanghai University of Sport has been authorized to access and collect these data via the specific data interface.

#### Subjects

All investigated samples were selected that met all of the following criteria from the NSFC database: (1) school type: public schools rather than private schools, considering the equity of government investment in education; (2) geographic scope: all primary schools in the studied region. The investigated region was Shanghai, which is a well-developed international city and consisted of 7 urban districts, 8 suburban districts, and 1 urban-suburban fringe district. The reason is that the primary educational resources and infrastructure in the city, conspicuously exhibit regional characteristics in suburban and urban districts, owing to the imbalance of population and economic development. Thus, the distribution and condition of SSFs in the city could be deemed as the epitome of the world. In detail, stipulated by the Law of the People’s Republic of China on Education, the subjects were allocated into three school categories (see Table 1): primary school (5-year curriculum, PS, n = 666), nine-year schools (Grade 1 to 9 combined, 9CS, n = 150) and twelve-year schools (Grade 1 to 12 combined, 12CS, n = 29). The inequalities in the distribution of samples emerged because all schools in the investigated region were studied in this paper. As for the geographical distribution pattern, the urban primary schools and the suburban were approximately equal in quantity with ratios of 1:1.07.

**Table 1:** Overview of primary education in Shanghai, China.

Primary School Category	Suburban	Urban	Total
12CS	13	16	29
9CS	89	61	150
PS	306	360	666
Total	408	437	845

12CS = 12-year school; 9CS = 9-year school; PS = 5-year school, primary school only.

#### School facility condition measurements

The statistical indicators of NSFC covered all aspects of sports facilities and were divided into 3 categories: (1) general information (ownership type, ownership details, address, and athletic team service information), (2) sports facility information (type, scale, number of staff, location type, year built, and open service), and (3) operational information (operation mode, hosted sports events, fitness training programs, operation income, and costs). The operational information referred to the utilization and operation of a particular school facility and relevant business. However, sports facilities in all samples in this paper were merely used to meet the demand of primary education for public benefit. Therefore, the operational information was insignificant to this paper and omitted, whereas the rest relating to this study were selected and categorized. The details of each descriptive measurement are described as follows:

(1) Variety. The variety was assessed by the aggregated numbers of sports facility types in each school. In the statistic caliber of NSFC, the types of SSFs were investigated listed in Table 2. The types of indoor, outdoor, and total facilities were computed separately, for the sake of describing the condition of SSFs precisely. Hence, we selected the types of indoor (IT), outdoor (OT), and total facilities (TT) as the indicators of PSSFC variety.

**Table 2:** Classification and types of school sports facilities (SSFs).

Category	Types of School Sports facilities	Types
**Outdoor**	stadium, track field, soccer pitch (futsal and 7-a-side pitch included), basketball court (3-a-side pitch included), volleyball court, badminton court, tennis court, swimming pool, American football/rugby pitch, hockey pitch, table tennis table, handball field, cricket pitch, gate ball court, baseball field, archery field, bocce courts, skating field, skateboarding/roller skating field, and fitness equipment	20
**Indoor**	track field, fitness room, basketball court, volleyball court, handball field, gymnastics room, badminton court, table tennis room, martial arts room, fight event training room, fitness room, yoga room, weightlifting room, fencing room, chess, and card room, bowling room, futsal pitch, tennis court, hockey pitch, archery field, equestrian field, ice hockey field, skating field, curling kettle field, skateboarding/roller skating field, squash room, and gateball room	27

(2) Size. The size of the SSFs was determined by the area of an individual facility. The area was defined as the effective accessible area used for training, competition, fitness activities, and safety protection. To demonstrate the variance of SSFs, the area of indoor (IA), outdoor (OA), total facilities (TA), and those average values were adopted as the indicators of PSSFC size.
(3) Location. The location (SL) of an individual school was used to differentiate whether an individual belongs to the urban or suburban category. However, the geographical data of SSFs in NSFC were described in form of addresses, which were descriptive and incapable of directly expressing the geographical attribution of each SSFs. Furthermore, as an exceptional case, the Pudong District (the second largest and unique urban-suburban fringe district in Shanghai) is allocated with the most fundamental education resources (4 times more than the largest Chongming District [20]). As an alternative, the individual’s SL in this paper was extracted via programming from the Baidu Map and calculated by measuring the distance of sampled school to the Building of Shanghai Municipal Peoples’ Government (City Hall) and computed by using the Haversine formula [21]. Thus, we divided all samples into urban (with a distance less than equivalent to 15 km, *N* = 385) and suburban (a distance greater than 15km, *N* = 460) in light of the General Development Plan of Shanghai City.

### Data processing and analysis

**Fig 1:**
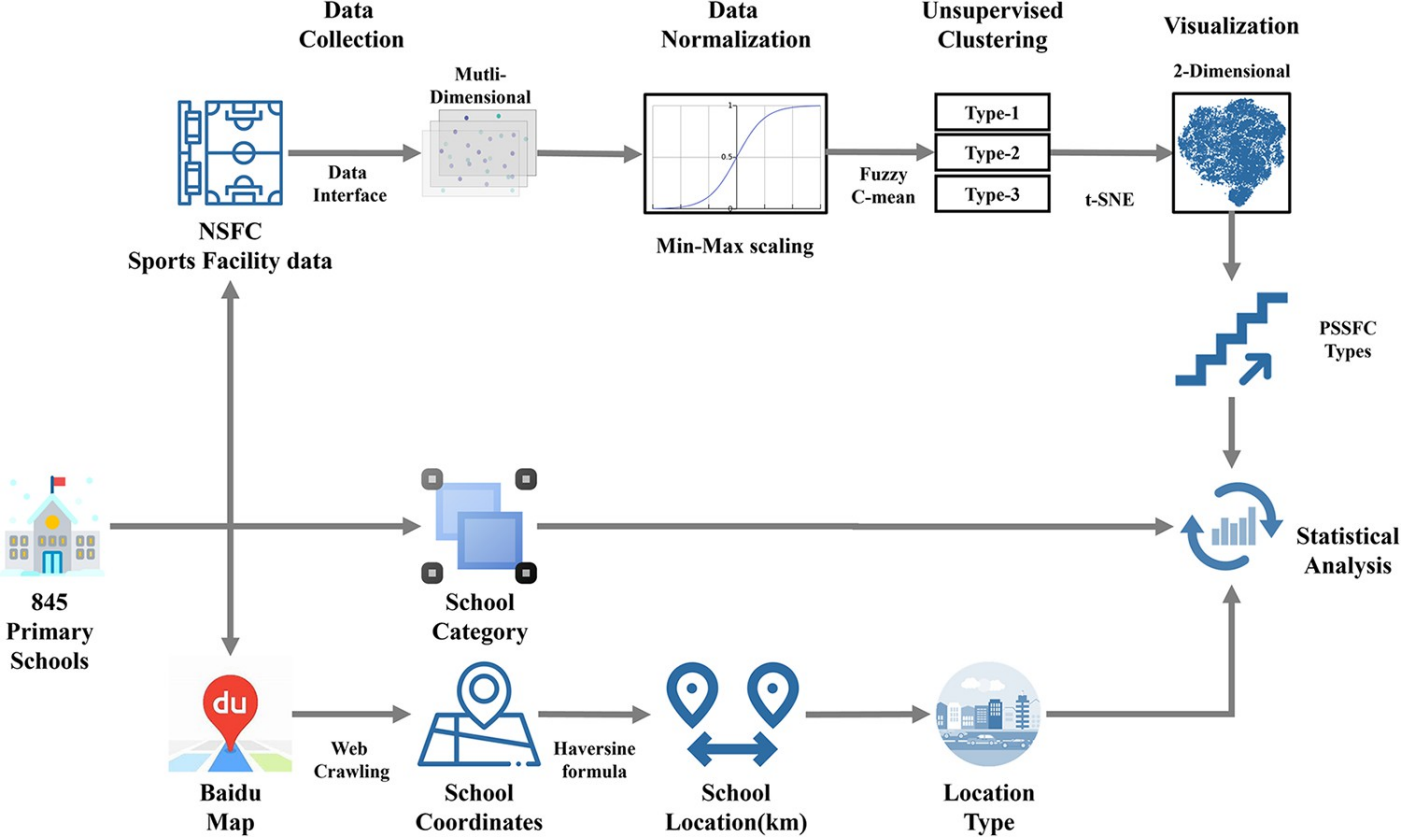
Overview of data processing and modeling pipeline on the assessment of PSSFC.

The general framework of analysis in this paper is depicted in Fig 1. All SSFs data were processed and modeled by using Python (Python 3.7.4 version for Windows, the Python Software Foundation, Delaware, USA). The location of each sample was extracted and consolidated with the PSSFC to structure a 9-dimensional SSFs dataset. The original SSFs dataset contained four numeric segments that featured the PSSFC overall (TT, TA, and Average TA), indoor (IT, IA, and Average IA), outdoor (ON, OA, and average OA), and SL. In common terms, the overall segment was the summation of indoor and outdoor, which performed a linear relation. Inversely, the relation between indoor and outdoor segments exhibited nonlinearity and independence. The min-max normalization was operated to scale the data between 0 and 1.

Clustering is usually applied to distinguish the objects with similar attributes by allocating them into different groups (clusters) [22] when the raw data are unlabeled. Based on the reality that no prior model or criteria for the classification of SSFC has been proposed, the solution shifts to rely solely on the features and similarity of SSFC as the criteria for attribute classification, which falls under the realm of unsupervised clustering. Most clustering algorithms were hard clustering (HC) [23,24]. Actually, in some cases, each data sample could present the belongingness to two or more clusters. Thus, the typical HCM algorithms, such as K-means and Hierarchical behaved with low applicability. Therefore, we introduced the Fuzzy-C Mean (FCM) Algorithm [25] to cluster primary schools by similarity in PSSFC and sort to certain levels according to the characteristics of each cluster. FCM has been regarded as one of the most popular and feasible clustering algorithms [22] to tackle the problems regarding MRI image segmentation [26–28], sentiment classification [29], power quality recognition [30], fault detection [31], disease detection [32,33]. It should be noted that despite the undoubted success of deep learning clusters in computer vision applications, for instance, DeepCluster [34] and DFuzzy [35], we adopted the traditional clustering method for the reason of simplicity and small scale of numeric PSSFC data.

FCM, generated from the fuzzy theory, optimizes the objective function to obtain the degree of membership (DM) for each sample point to all class centers in the interval 0 to 1 (soft clustering) rather than binary values 0 or 1 (HC). As a result, each sample can be distributed to multiple clusters by computing its DM to each cluster. In this issue, given the embedded SSFs dataset *X* = {*x*_1_, *x*_2_,…, *x*_n_} (in the two-dimensional space after the process of *t*-SNE) and *j* clustering centers *C* = {*c*_1_, *c*_2_,…, *c*_*j*_}, a 2*×n* matrix *U* can be generated to describe the clustered result:

$$U=(\begin{array}{lll} u_{11} & \cdots & u_{1j}\\ u_{21} & \ldots & u_{2j} \end{array}),$$ (1)

where *u*_*ij*_ ranging from zero to one is the DM for an individual *x*_*i*_ belonging to category *j*, which can be calculated by:

$$u_{ij}=\frac{1}{\sum_{k=1}^{c}{\left(\frac{\left\|x_{i}-c_{j}\right\|}{\left\|x_{i}-c_{k}\right\|}\right)}^{\frac{2}{m-1}}},\;c_{j}=\frac{\sum_{i=1}^{N}u_{ij}^{m}x_{i}}{\sum_{i=1}^{N}u_{ij}^{m}},$$ (2)

where *m* is a real number greater than 1. After the *t* times iteration, FCM attempts to minimize the cost function as below:

$$J_{m}=\sum_{i=1}^{N}\sum_{j=1}^{c}u_{ij}^{m}{\left\|x_{i}-c_{j}\right\|}^{2},1\leq m<\infty .$$ (3)

FCM terminates until the following condition is compromised:

$$\max_{ij}\;\{\left|u_{ij}^{\left(t+1\right)}-u_{ij}^{\left(t\right)}\right|\}<\varepsilon ,$$ (4)

which implies that the result has achieved a comparative (local or global) optimum, and the DM will not be altered significantly if the iteration continues.

Generally, the numbers of cluster center *c* in FCM are predetermined parameters [36]. However, a lack of standards or guidance for evaluating PSSFC has been an issue for the current studies. Therefore, the numbers of the cluster center were adjusted from one to ten to explore the best classification of SSFs. As for the configuration of FCM, the fuzzy partition coefficient (FPC) [37] was selected as the validity index of clustering shown in Eq (5), and the other parameters [29] were selected as *m* = 1.5, *t* = 1000, and *ε* = 0.001.


$$FPC=\frac{\frac{1}{c}\sum_{i=1}^{N}\sum_{j=1}^{c}u_{{}_{ij}}^{2}-1/c}{1-1/c}$$ (5)

To visualize 9-dimensional SSFs data, *t*-stochastic neighbor embedding (*t*-SNE) [38,39], was applied to visualize PSSFC in the lower dimension. The *t*-SNE is has been proved as a visualization approach for non-linear high-dimensional data with high efficiency and widely used in bioinformatics [40,41], disease assessment [42], signal processing [43], fault identification [44], and other domains. It should be also clarified that notwithstanding the complexity scaling (*O*(n^2^)) of *t*-SNE is higher than PCA and LDA, *t*-SNE has an excellent performance in the visual description of SSFs features in primary schools, on account of the finite numbers of measurements and samples. The configuration of the parameters, the perplexity *perp* and times of iteration *t*, is an inevitable issue to be tackled when applying the *t*-SNE algorithm.

The original data were labeled with the different types (Type-1, Type-2, and Type-3) after the processing of FCM and *t*-SNE. The above three types of PSSFC were exhibited with descriptive statistics (means and standard errors, mean ± SE). Furthermore, a two-way ANOVA was performed to figure out the differences in the variety and size in each PSSFC type adjusted for the school category and location of primary schools, with the significance level set at 0.05. All analyses were processed using the Statistical Package for the Social Sciences (SPSS) 22.0 on Windows (IBM, Chicago, IL, USA).

## Results

### Clustering and visualization of PSSFC

#### Result of unsupervised clustering

**Fig 2:**
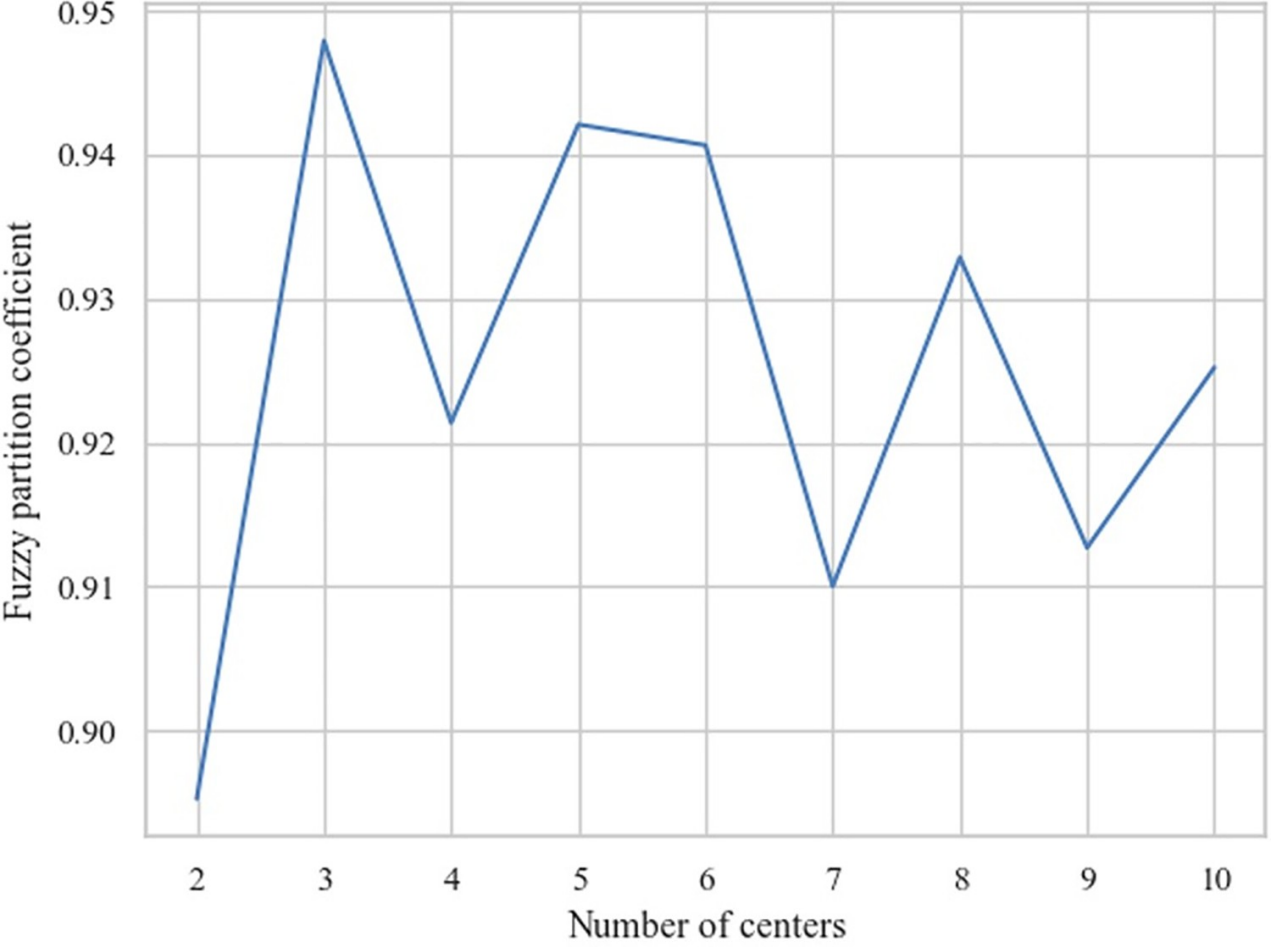
The comparison of the fuzzy partition coefficient (FPC) for the selecting centers of FCM.

Fig 2 presents the results of FCM clustering on the embedded PSSFC via *t*-SNE. The samples clustered into the same level were marked with the same color. To select the optimal *c*, we compared the FCM clustering effect when c ranged from 2 to 10. it could be recognized from Fig 4 that when *c* = 3, the clustering result conformed to human beings’ intuition. To further prove that *c* = 3 is the optimal clustering center, Fig 5 exhibits the DM for an individual sample to each clustered level when *c* = 3 and FPC reached the summit (FPC = 0.9479).

**Fig 3:**
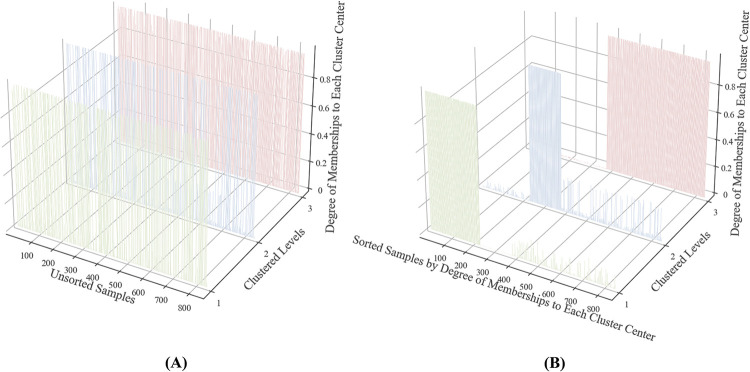
The degree of membership of PSSFC for each primary school clustered by FCM (*c* = 3). (1) All sampled schools in the initial order and random sort. (2) Sorted by the DM for each school belong to every cluster.

In Fig 3, we adopted the different colors to represent the DM for a particular sample to different levels. As mentioned above, the DM indicates that the strength of belongingness for an individual sample to a clustering level in fuzzy clustering. the Panel (1) in Fig 3 shows the DM for each PSSFC with the initial sequence, where the *x*-axis is the order number of samples, the *y*-axis is the order number of the cluster center, and the z-axis is the DM *u*_*ij*_ of sample *i* belonged to cluster center *j*. For a better demonstration of the feasibility of FCM, the order of the original data is sorted and numbered according to the individual’s corresponding DM to a certain level from the largest to the smallest, while the *y*-axis and *z*-axis are unchanged, the *x*-axis is converted to the sorted sequence. The results show that all samples were explicitly li allocated into three independent categories by FCM.

#### Visualization of clustered PSSFC in two-dimensional space

**Fig 4:**
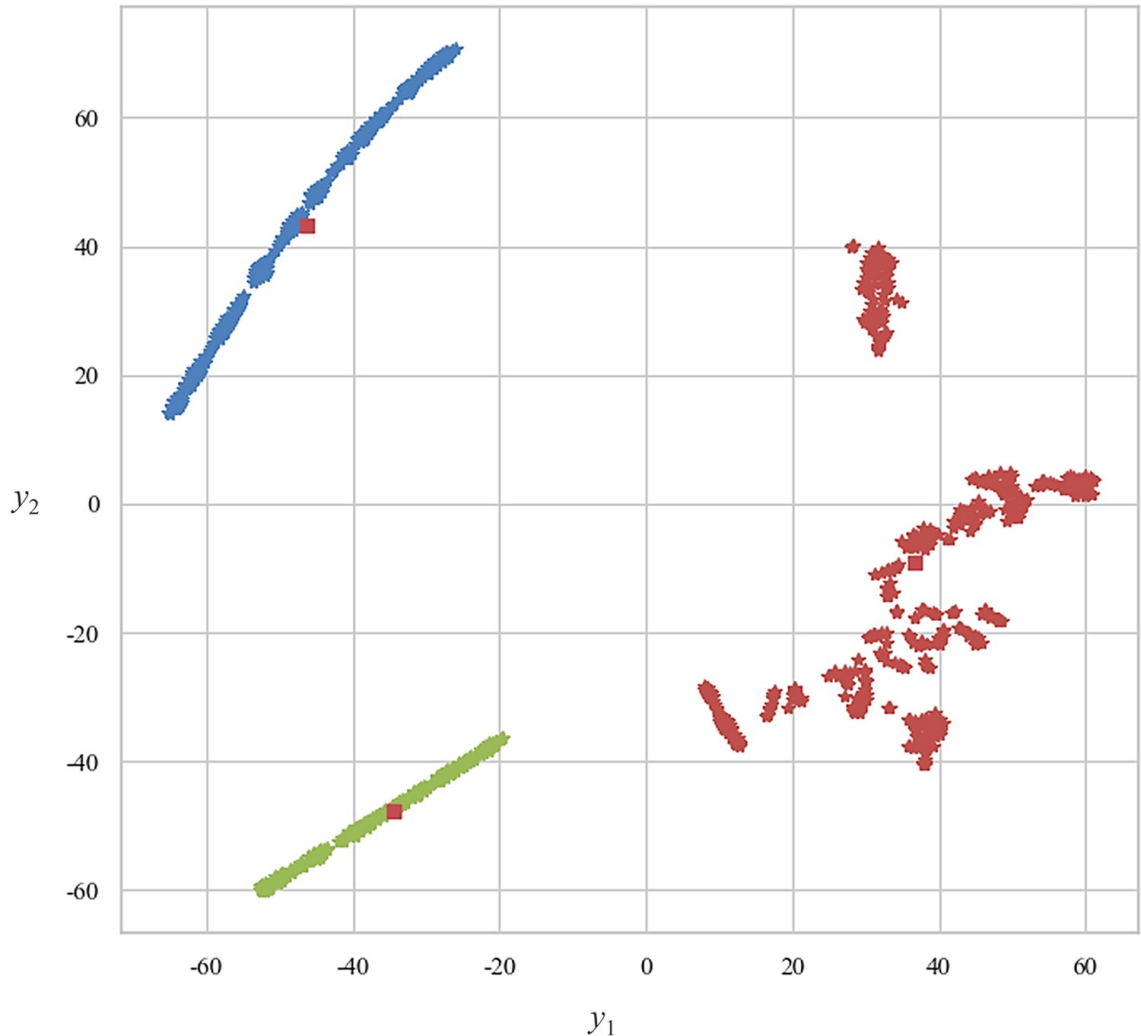
Embedding of clustered PSSFC into two dimensions via *t*-SNE.

Fig 4 shows the visual results of embedding PSSFC into the two-dimensional space by using *t*-SNE (*perp* = 15, *t* = 5000, and *η* = 200), the space (y1, y2), for all of the 845 sampled primary schools. Data with dissimilarity were isolated in the embedded space, while the samples of similar attributes were almost assembled into the adjacent area. This hinted that the utilization of *t*-SNE possesses the effectiveness of visualization on this issue and the capability to observe the global overview of clustered PSSFC visually.

### Descriptive characteristics of PSSFC at different levels

Table 3 exhibits the description and distribution of PSSFC with 3 types in detail generated by the defuzzification of the original dataset. In general, over half of the sampled schools were categorized to Type-3 (n = 463, 54.79%) while Type-2 of PSSFC was 17.52% (n = 148), and Type-1 schools occupied over a quarter. From the perspective of descriptive statistics, it could be concluded from Table 3 that the three categories showed a gradation in the overall PSSFC, the discernable characteristics of Type-3 schools were the deployment with at least 1 indoor facility in the total 3 sports facilities the diversity, and maximum area of sports facilities on average. In contrast to Type-1, the Type-2 and Type-3 schools showed the scarcity in the indoor facility, which indicated the fewer alternatives in the above schools when it suffered from inclement weather or air contamination. In addition, the individuals in Type-2 possess almost twice as large and quantitative as those in Type-1 in total facilities.

**Table 3:** Descriptive statistics of classified PSSFC by using machine learning (n = 845).

	Type-1	Type-2	Type-3
**Schools, n (%)**	234 (27.69%)	148 (17.52%)	463 (54.79%)
**Total Facilities**		
TT (n)	1.00±0.00	2.00±0.0	3.57±1.38*+
TA (*m*^2^)	1998.91±1539.69*	3815.92±2185.39*+×	6289.52±5030.98*+×
Average TA (*m*^2^)	1998.91±1539.69*	1907.96±1092.69*+×	1736.41±1143.34*+
**Indoor Facilities**		
IT (n)	0	0	1.09±0.76+
IA (*m*^2^)	0	0	549.9±585.67*+×
Average IA (*m*^2^)	0	0	432.43±374.57*+
**Outdoor Facilities**		
OT (n)	1.00±0.00	2.00±0.0	2.48±1.24*+
OA (*m*^2^)	1998.91±1539.69*	3815.92±2185.39*+×	5739.62±4776.73*+×
Average AO (*m*^2^)	1998.91±1539.69*	1907.96±1092.69*+×	2397.4±1666.11*+

Data are expressed as mean ± SE.

* significant difference between urban and suburban primary schools at the same PSSFC level (p*<*0.05).

^**+**^ significant difference among different school categories (PS, 9CS, 12CS) at the same PSSFC level (p*<*0.05).

^**×**^ significant interaction of the types and location of schools between the PSSFC variables at the same PSSFC level (p*<*0.05).

### Geographical and typological characteristics of PSSFC

#### Impact of location and school category on PSSFC

In this paper, the school location (L) and the school category (SC) were selected as the test factors. The effects of two factors and the interaction on PSSFC in intraclass schools were respectively analyzed, and the results of which are marked in Table 3. Table 4 indicates that the school location, the school category, and the interaction of the two had significant interclass effects on the performance of PSSFC when using the two-way ANOVA with a 0.95 significance level.

**Table 4:** Relationships between school location and school category and PSSFC.

Type	Variables	Source	*df*	SS	M	F	*p*-value
Type-1	TT (n)	L	1	0.007	0.007	1.539	0.216
		SC	2	0.000	0.000	0.028	0.972
		L×SC	2	0.001	0.000	0.060	0.942
	TA (m^2^)	L	1	2.22×10^7^	2.22×10^7^	5.162	**0.02401**
		SC	2	6.80×10^6^	3.40×10^6^	0.791	0.455
		L×SC	2	7.54×10^6^	3.77×10^6^	0.878	0.417
	Average TA (m^2^)	L	1	1.40×10^7^	1.40×10^7^	5.176	**0.02382**
		SC	2	6.88×10^6^	3.44×10^6^	1.268	0.283
		L×SC	2	8.56×10^6^	4.28×10^6^	1.577	0.209
	OT (n)	L	1	0.007	0.007	1.539	0.216
		SC	2	0.000	0.000	0.028	0.972
		L×SC	2	0.001	0.000	0.060	0.942
	OA (*m*^*2*^)	L	1	2.22×10^7^	2.22×10^7^	5.162	**0.02401**
		SC	2	6.80×10^6^	3.40×10^6^	0.791	0.455
		L×SC	2	7.54×10^6^	3.77×10^6^	0.878	0.417
	Average OA (*m*^*2*^)	L	1	1.40×10^7^	1.40×10^7^	5.176	**0.02382**
		SC	2	6.88×10^6^	3.44×10^6^	1.268	0.283
		L×SC	2	8.56×10^6^	4.28×10^6^	1.577	0.209
Type-2	TT (n)	L	1	0.005	0.005	0.718	0.398
		SC	2	0.039	0.019	2.975	0.054
		L×SC	2	0.021	0.010	1.588	0.208
	TA (*m*^*2*^)	L	1	1.48×10^8^	1.48×10^8^	36.143	**1.44×10** ^**−8**^
		SC	2	1.18×10^8^	5.90×10^7^	14.409	**1.97×10** ^**−6**^
		L×SC	2	6.50×10^7^	3.25×10^7^	7.932	**5.40×10** ^**−4**^
	Average TA (*m*^2^)	L	1	3.46×10^7^	3.46×10^7^	39.285	**4.01×10–09**
		SC	2	2.37×10^7^	1.19×10^7^	13.444	**4.44×10** ^**−6**^
		L×SC	2	1.34×10^7^	6.69×10^6^	7.581	**7.40×10** ^**−4**^
	OT (n)	L	1	0.005	0.005	0.718	0.398
		SC	2	0.039	0.019	2.975	0.054
		L×SC	2	0.021	0.010	1.588	0.208
	OA (*m*^*2*^)	L	1	1.48×10^8^	1.48×10^8^	36.143	**1.44×10** ^**−8**^
		SC	2	1.18×10^8^	5.90×10^7^	14.409	**1.97×10** ^**−6**^
		L×SC	2	6.50×10^7^	3.25×10^7^	7.932	**5.40×10** ^**−4**^
	Average OA (*m*^*2*^)	L	1	3.46×10^7^	3.46×10^7^	39.285	**4.01×10–09**
		SC	2	2.37×10^7^	1.19×10^7^	13.444	**4.44×10** ^**−6**^
		L×SC	2	1.34×10^7^	6.69×10^6^	7.581	**7.40×10** ^**−4**^
Typel-C	TT (n)	L	1	63.336	63.336	38.275	**1.37×10–09**
		SC	2	52.001	26.000	15.713	**2.52×10** ^**−7**^
		L×SC	2	8.565	4.283	2.588	0.076
	TA (m^2^)	L	1	1.69×10^9^	1.69×10^9^	100.465	**1.69×10** ^**−21**^
		SC	2	1.59×10^9^	7.96×10^8^	47.377	**2.04×10–19**
		L×SC	2	3.39×10^8^	1.70×10^8^	10.085	**5.18×10** ^**−5**^
	Average TA (*m*^*2*^)	L	1	5.50×10^7^	5.50×10^7^	64.860	**7.11×10** ^**−15**^
		SC	2	4.32×10^7^	2.16×10^7^	25.444	**3.35×10** ^**−11**^
		L×SC	2	1.15×10^6^	5.76×10^5^	0.679	0.508
	IT (n)	L	1	0.829	0.829	1.521	0.218
		SC	2	12.658	6.329	11.614	**1.20×10** ^**−5**^
		L×SC	2	0.746	0.373	0.685	0.505
	IA (*m*^*2*^)	L	1	1.20×10^6^	1.20×10^6^	4.203	**4.09×10** ^**−2**^
		SC	2	2.33×10^7^	1.17×10^7^	40.806	**5.01×10** ^**−17**^
		L×SC	2	3.39×10^6^	1.70×10^6^	5.936	**2.85×10** ^**−3**^
	Average IA (*m*^*2*^)	L	1	5.74×10^5^	5.74×10^5^	4.410	**3.63×10** ^**−2**^
		SC	2	4.37×10^6^	2.18×10^6^	16.765	**9.43×10** ^**−8**^
		L×SC	2	2.45×10^5^	1.23×10^5^	0.941	0.391
	OT (n)	L	1	78.654	78.654	58.283	**1.35×10–13**
		SC	2	16.130	8.065	5.976	**2.74×10** ^**−3**^
		L×SC	2	4.277	2.139	1.585	0.206
	OA (m^2^)	L	1	1.60×10^9^	1.60×10^9^	105.085	**2.52×10** ^**−22**^
		SC	2	1.27×10^9^	6.33×10^8^	41.599	**2.56×10** ^**−17**^
		L×SC	2	2.91×10^8^	1.45×10^8^	9.552	**8.64×10** ^**−5**^
	Average OA (*m*^*2*^)	L	1	6.43×10^7^	6.43×10^7^	28.261	**1.66×10** ^**−7**^
		SC	2	8.09×10^7^	4.04×10^7^	17.763	**3.73×10** ^**−8**^
		L×SC	2	4.08×10^5^	2.04×10^5^	0.090	0.914

The school location (L) and school category (SC) of PSSFC was selected the impactors with and analyzed by two-way ANOVA with the significance level set at 0.05. TT = total types of PSSFC; TA: total area of PSSFC; IT = types of indoor PSSFC; IA = area of indoor PSSFC; OT = types of outdoor PSSFC; OA = area of outdoor PSSFC; L = school location; SC = school category; L × SC = location and school category; SS = sum of squares; *df* = degree of freedom; and MS = mean squares.

In Tables 3 and 4, the findings revealed that the individual’s location was the major influence on its PSSFC. Specifically for each clustered type, the suburban Type-1 schools, which were configured with 1 outdoor facility and possessed larger size in PSSFC than the urban of the same type (*F* = 5.162, *p*<0.05). In the Type-2 schools with outdoor facilities, the numbers of PSSFC (TT and OT) had no significant relationship with the individual’s location (*p*>0.05), however, the location index significantly related to the scale of outdoor facilities in OA (*F* = 36.143, *p*<0.01) and Average OA (*F* = 39.285, *p*<0.01). The majority of PSSFC indicators in the Type-3 schools except IT (*p*>0.05) was significantly associated with the school location, especially in TA (*F* = 100.465, *p*<0.01), Average TA (*F* = 64.850, *p*<0.01), OT (*F* = 58.283, *p*<0.01) and OA (*F* = 105.085, *p*<0.01). In conclusion, the suburban schools possessed better PSSFC than the urban in the aspect of scale.

The school category did not significantly relate with the Type-1 schools but was yet another crucial affecting factor of PSSFC in Type-2 and Type-3 schools. More precisely, all of the PSSFC variables (*p*<0.05) in the Type-3 schools were associated with the relating school category, which determined the indoor indicators, IT (*F* = 11.614, *p*<0.01), IA (*F* = 40.806, *p*<0.01), and IA Average (*F* = 16.765, *p*<0.01) of indoor PSSFC in these schools. The scale of outdoor PSSFC, in the Type-2 schools, OA (*F* = 14.409, *p*<0.01) and Average OA (*F* = 13.444, *p*<0.01), had a significant link with the school category. However, when it referred to Type-1, the school category had no significant impact on those PSSFC (*p*>0.05).

Considering the interaction of school location and school category, these two factors had major impacts on the TA (*F* = 10.085, *p*<0.01), OA (*F* = 9.552, *p*<0.01), and IA (*F* = 5.936, *p*<0.01) of Type-1 and the average outdoor space of Type-2 (*p*<0.05). However, no significant impact on PSSFC in the Type-1 schools was found (*p*>0.05).

The analytic results indicate that the location (suburban or urban) of primary school was associated with types (except Type-2) and area (both aggregated and averaged) of overall and outdoor facilities, rather than the types of indoor facilities. Furthermore, the suburban schools (especially in those covering more grades) were resourced with the larger space and more multiplicity of PSSFC, to accommodate more schoolchildren to participate in physical activities than the urban.

### Geographical and typological distribution

Table 5 shows the comparison of PSSFC types in quantity and percentage in respect of the spatial and phyletic distribution. Combined with the above-mentioned results, over half of the primary schools were constructed with well-conditioned sports facilities (Type-3, the suburban and urban is 56.37% and 53.32% respectively). However, the proportion of Type-1 schools in the inner city (32.72%) was higher than those in the suburban districts (22.31%) by over 10%. Another notable finding was that the PSSFC in PS occurred to be inferior to those in 9CS and 12CS, according to the percentage of those Type-1 (35.83% of the urban PS and 27.78% of the suburban).

**Table 5:** Numeric statistics on 3 types of PSSFC clustered by using machine learning.

Location	PSSFC		
	Type-1	Type-2	Type-3
**Suburban (n = 408), n (%)**	91(22.31%)	87(21.32%)	230(56.37%)
12CS, n (%)	(0%)	(0%)	13(100%)
9CS, n (%)	6(6.74%)	14(15.73%)	69(77.53%)
PS, n (%)	85(27.78%)	73(23.85%)	148(48.37%)
**Urban (n = 437), n (%)**	143(32.72%)	61(13.96%)	233(53.32%)
12CS, n (%)	1(6.25%)	1(6.25%)	14(87.50%)
9CS, n (%)	13(21.31%)	6(9.84%)	42(68.85%)
PS, n (%)	129(35.83%)	54(15.00%)	177(49.17%)

12CS = 12-year school; 9CS = 9-year school; PS = 5-year school, primary school only.

**Fig 5:**
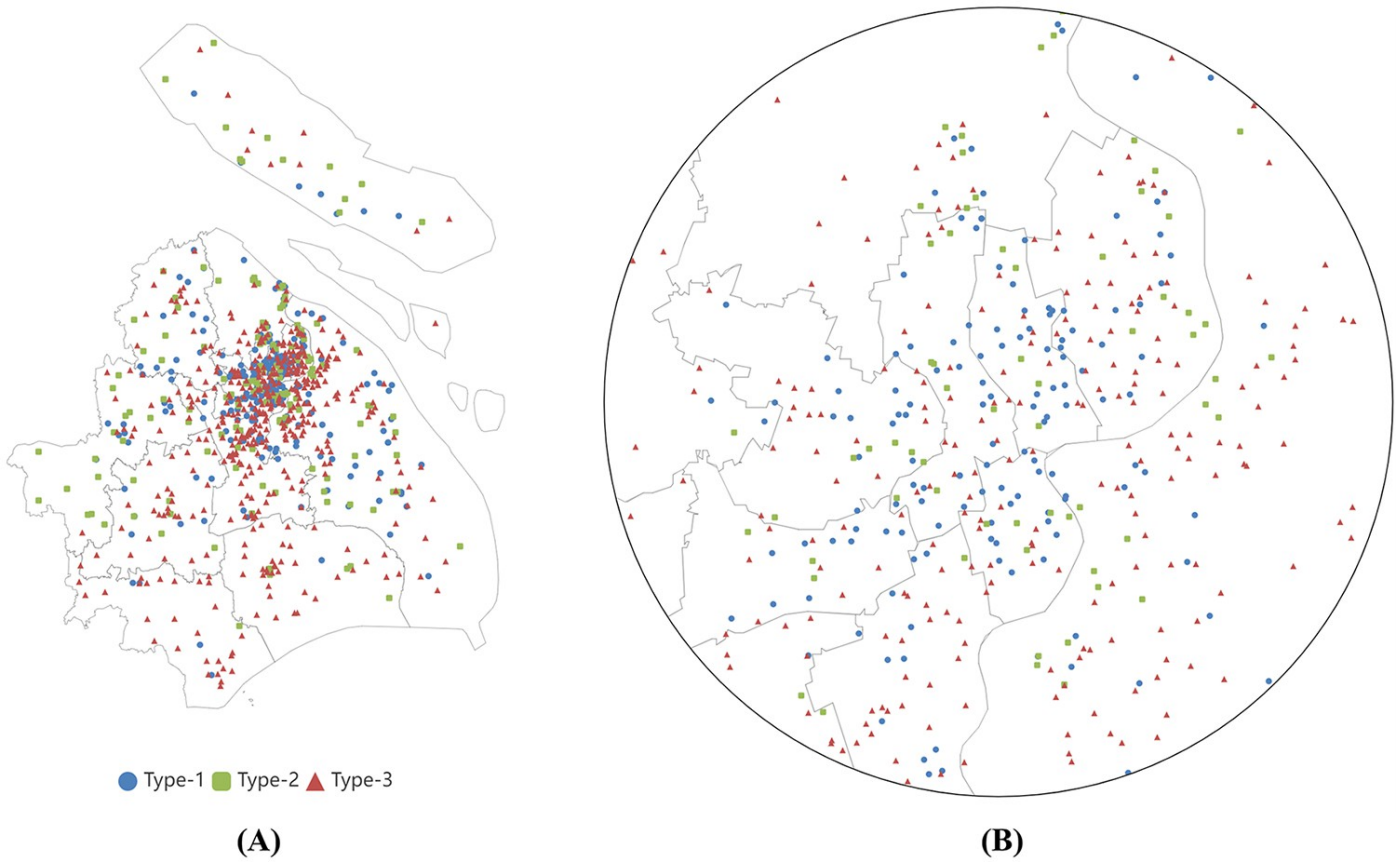
Geographical distribution of PSSFC with three categories in Shanghai. **(A) The geographical distribution panorama of PSSFC in Shanghai.** (B) The distribution of Type-1 PSSFC in Shanghai. (C) The distribution of Type-2 PSSFC in Shanghai. (D) The distribution of Type-3 PSSFC in Shanghai.

**Fig 6:**
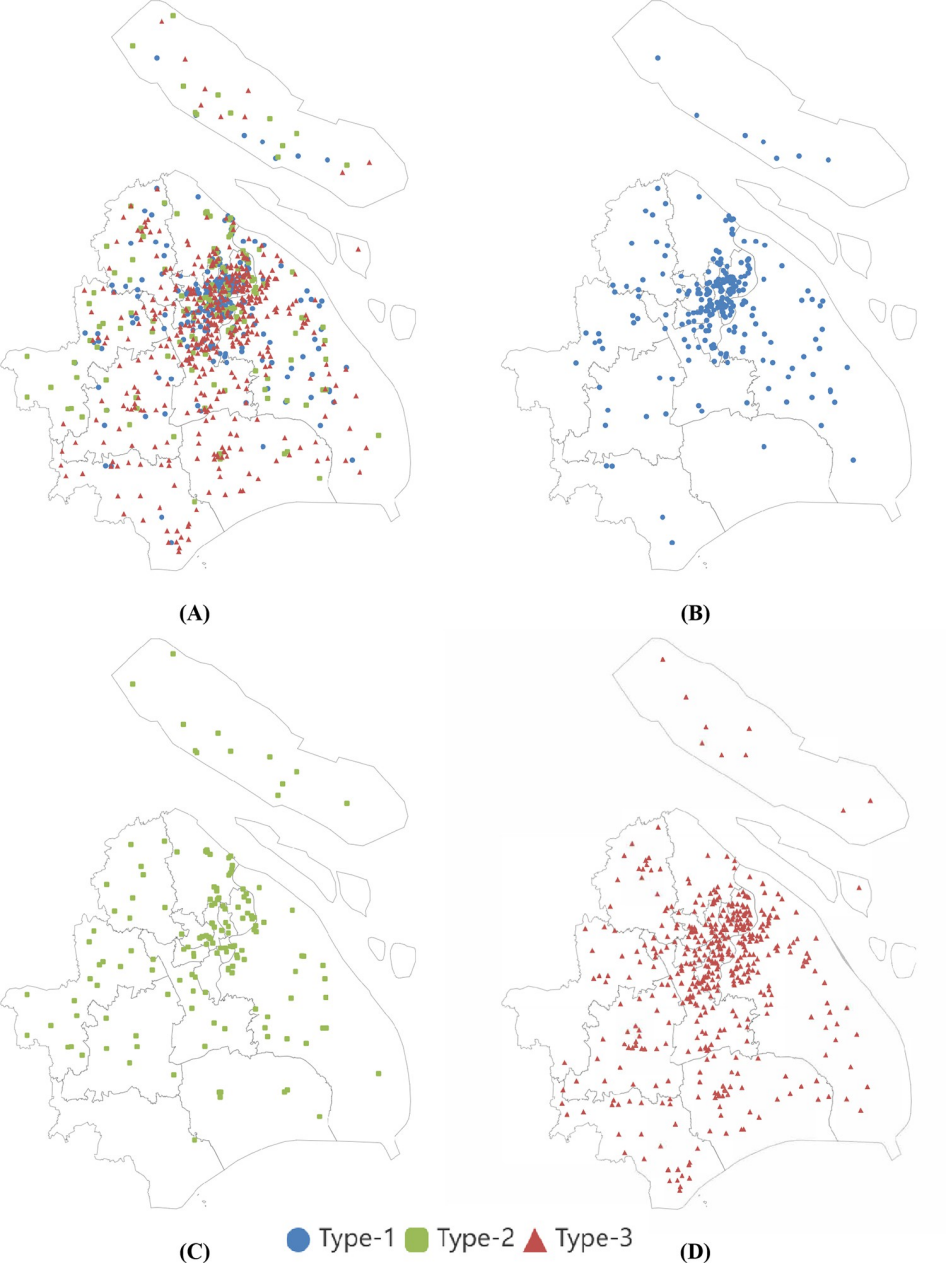
Geographical distribution of f PSSFC with three categories in urban districts in Shanghai by zoomed in to the scope of the urban area (distance ≤15 kilometers to the Shanghai Municipal Peoples’ Government).

Fig 5 demonstrates the color-separated geographical scatter diagram, to observe the PSSFC in the scope of geographical distribution and illuminate the impact of location on PSSFC. Panel (A) illustrates the distribution of schools with clustered marks within the investigated region on the administrative map, and Panel (B) to Panel (D) presents the distribution of schools at a particular type respectively. The PSSFC levels of samples were marked by red, green, and blue, which is in line with Fig 4. Moreover, the distribution of schools within 15 km of the City Hall at all levels was listed in Fig 6 to give a clearer picture of the downtown PSSFC. It could be summarized from Figs 5 and 6 that the distribution of primary schools in the researched region showed a feature of outward diffusion from a central point. The number of primary schools in the urban area is strikingly more than that of those in the suburban. In other words, the urban contained more abundant resources for primary education. Nevertheless, the distribution of PSSFC stands out in stark contrast to the aforementioned situation: the better the school conditions were, the more uniform the geographical distribution presents; the worse the school conditions were, the more geographical distribution was concentrated in the urban area.

## Discussion

Prior studies have concerned the scientific and subjective assessment of PSSFC when investigating the correlation between children’s PA and PSSFC. As a supplement, this study sought to propose an innovative and objective method for evaluating PSSFC via machine learning. Our findings demonstrate that the unsupervised machine learning approach on this issue performed with high interpretability and visuality in differentiating PSSFC characteristics. The results of clustering PSSFC into three types comprehensively depict the interclass differences and characteristics of PSSFC. Our experiment extends the present studies on the multidimensional analysis of PSSFC methodologically.

In the study, the location, numbers of grade levels, school category, numbers, and square meters of sports facilities were considered as evaluation indicators of PSSFC, in line with previous studies [13,17,18,45]. The difference was that the data used in this study were collected from not the questionnaire but the official statistics. Among the above, the space for children’s PA had a positive correlation with both the duration and intensity of physical activity [46–48]. The type of sports facilities influenced students’ activity level by gender: boys dominated outdoor sports facilities, such as the soccer pitch, with girls preferring indoor activities [49], such as dancing and gymnastics [50]. As seen in Table 3, the quantified evaluation on PSSFC was consistent with the above finding, which suggests that a primary school with PA-demand-compliant PSSFC should be configured with 2 types of sports facilities (including 1 indoor preferred) and at least 2000 m^2^ accessible area. Besides, the indoor PSSFC performed as a significant indicator: primary schools with at least 1 indoor facility might promote children’s MVPA, even in recess time [51]. When it refers to those without indoor facilities, the types and square meters of outdoor PSSFC were the decisive indicators for the reason that outdoor sports condition of primary school had an underlying impact on schoolchildren’s physical activities and health [18,52,53]. The above-mentioned findings identified the effectiveness and validity of unsupervised machine learning.

Regarding the impact of location and school category, the analytical results in Table 4 indicated that PSSFC has a striking association with the individual location, especially for the aggregated and outdoor sports space. The similarity was proved in Norway [13,14], Taiwan [45], and Canada [54]. Although the urban PSSFC was probably affected by the shortage of land resources in developed cities and seemed to be inferior to the PSSFC in suburbs, the high density of inner-city sports facilities, particularly indoor facilities, supplements the accessibility of children’s PA [55–58]. To the best of our knowledge, it is identified that the significant links between PSSFC variables and individual school categories. In the usual sense, as the most populous type among all three types, 12CS catered to 7–12 years old students and required the best PSSFC for multigrade-shared PA in recess and after school. A possible explanation for these results may be relevant regulation of primary education in mainland China [59]. It stipulates that a PA room, greater than 40 m^2^, must be deployed in urban 9CS and 12CS, which is not the essential requirement of PS. This explains the association between indoor facilities area and school categories.

While providing new insights into the innovation of PSSFC assessment, several limitations of this study should be clarified. Private schools, which are the crucial part of primary education, were not involved. The omission of these schools may have resulted in some potential bias, although it is difficult to make assertions on the direction and size of this bias. Furthermore, although the geographic factors of urban and suburban areas were considered, the influence of geomorphology on the layout of primary school was not taken into account. The results are specific to primary schools in mainland China, and as such are likely to be context-specific. Moreover, the analysis is representative and cross-sectional in the developed region, which may not apply to the rest of world. Further research in different countries among different landforms would be beneficial. The approach of visualization and evaluation on PSSFC is exclusively based on objective data. Due to the lack of more detailed indicators in the NSFC, the following potential factors should be involved in further practical applications and studies: subjective assessment, the student population in each school, PA space per student, and land occupation and floor space of each school. Last but not least, constrained by the lack of data and the restriction of any experiment in primary schools induced by the COVID-19 pandemic, this study was barely limited to investigating the PSSFC without the analysis of results with the students’ PA and PE the in sampled schools. Despite these limitations, this study provides a demonstration of interdisciplinary research between artificial intelligence and the PA environment and analyzes the spatial and phyletic characteristics of PSSFC in developing countries. The evaluation method on PSSFC can be used to inform the regionalized and personalized intervention for children’s PA promotion, according to the current state of individual PSSFC.

## Conclusions

The finding in this paper verified that combined-approach unsupervised learning facilitates understanding the characteristics of sports facilities in primary schools in a visual way. The approach used in this study had acceptable levels of interpretability and was found to effectively differentiate sports facilities in primary schools. In addition, with the statistical analysis of the classification results, the spatial and typological association with the variables of sports facilities in primary schools was discovered and was consistent with previous studies. Our findings reveal that the regional school sports facility condition in primary schools has geographical distribution characteristics. Primary schools with more grades of students are equipped with more types and sizes of sports facilities.

Further work is aimed to quantify the PSSFC association with more varied factors, such as subjective remarks, attending numbers of students, and other potential influences.

## Supporting information

S1 Fig Comparison of classified overall PSSFC by school type and location. (TIF)

S2 Fig Comparison of classified indoor PSSFC by school type and location. (TIF)

S3 Fig Comparison of classified outdoor PSSFC by school type and location. (TIF)

S1 Dataset Data of sampled PSSFC. (PDF)

S1 File (CSV)
