# Supplementary figures and images for: An unsupervised machine learning approach to evaluate sports facilities condition in primary school
Source: PLoS One. 2022 Apr 20;17(4):e0267009. doi: 10.1371/journal.pone.0267009 (PMC9020747; doi:10.1371/journal.pone.0267009)

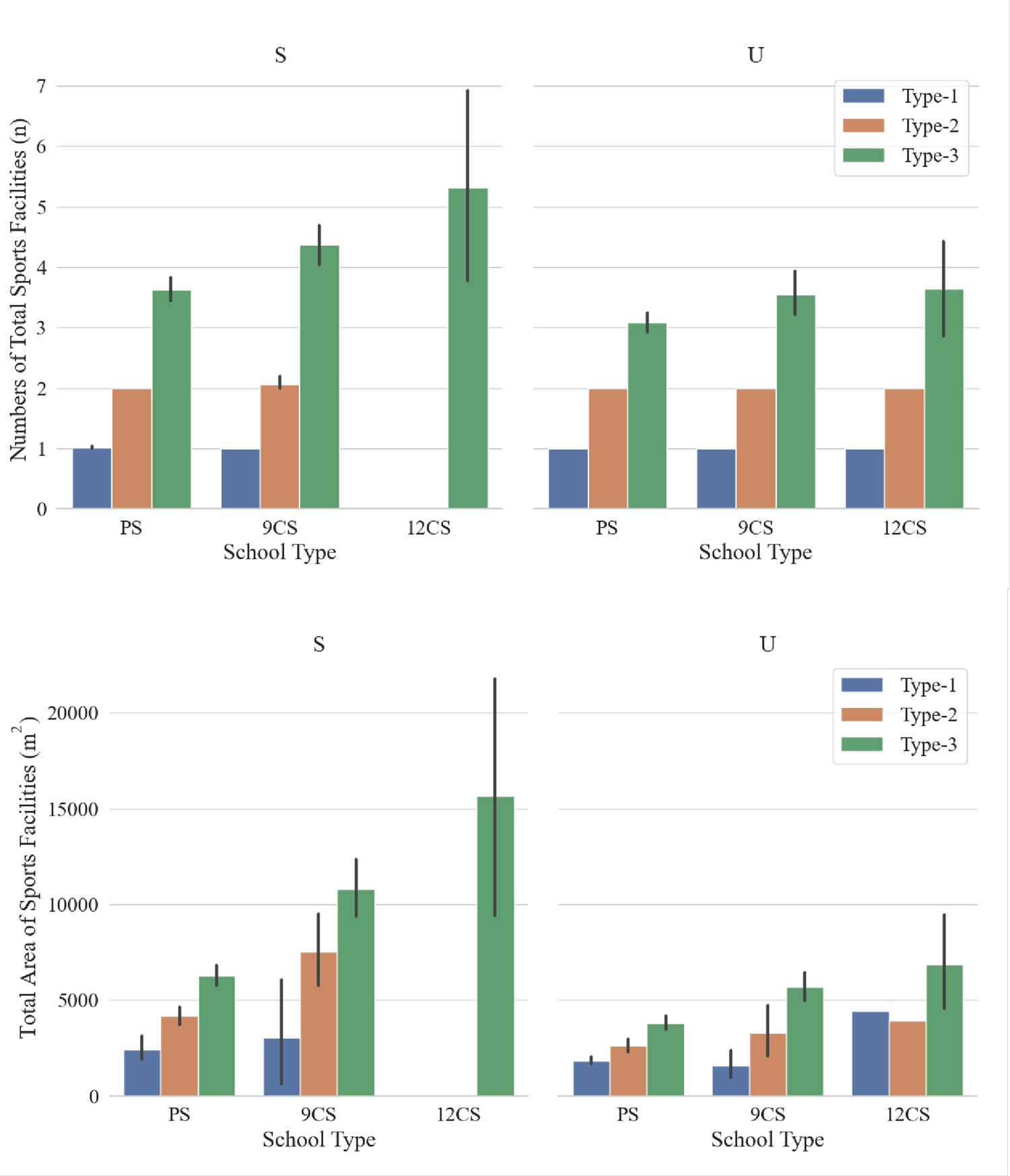

Supplement: S1 Fig — (TIF) [file pone.0267009.s001.tif]

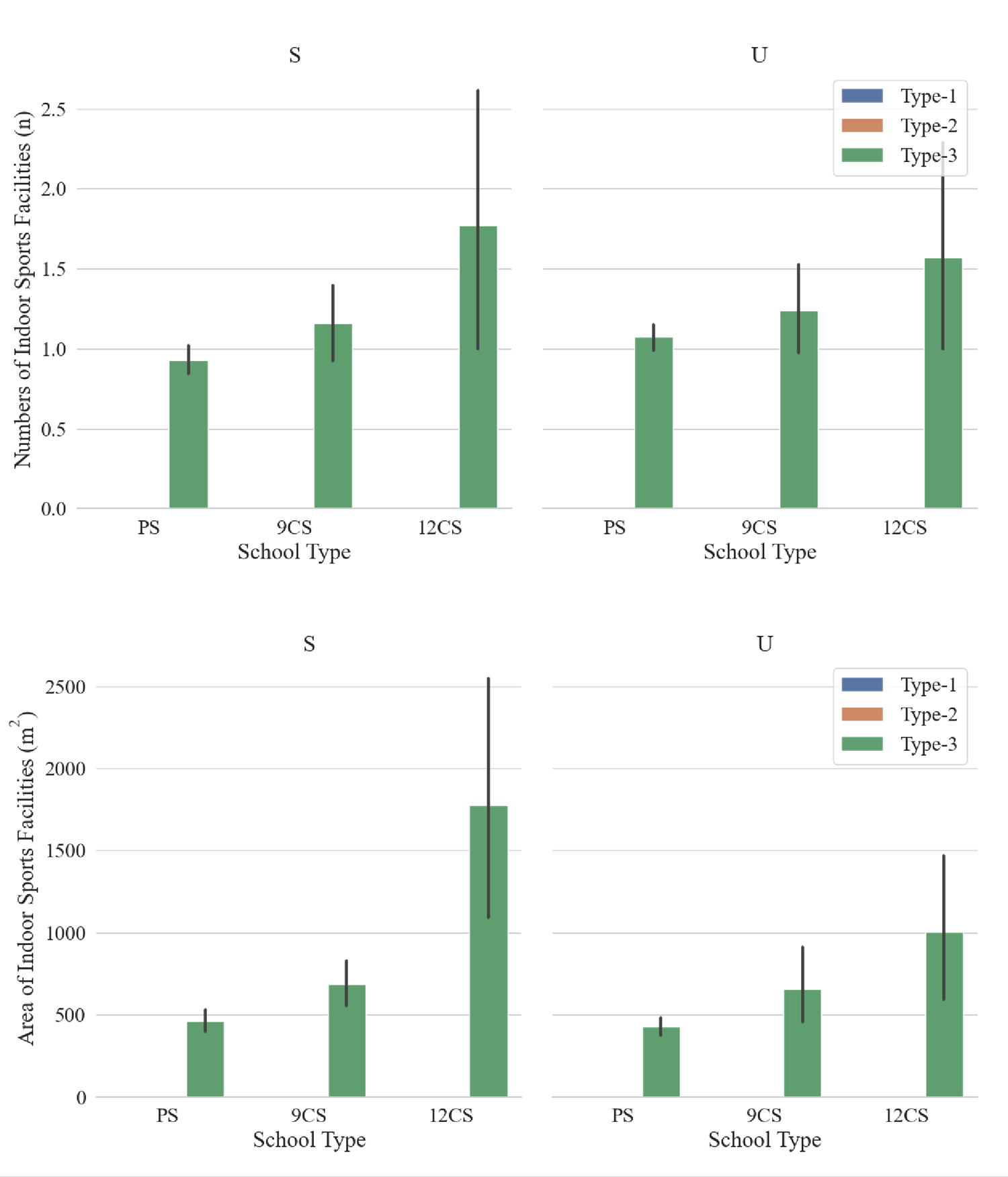

Supplement: S2 Fig — (TIF) [file pone.0267009.s002.tif]

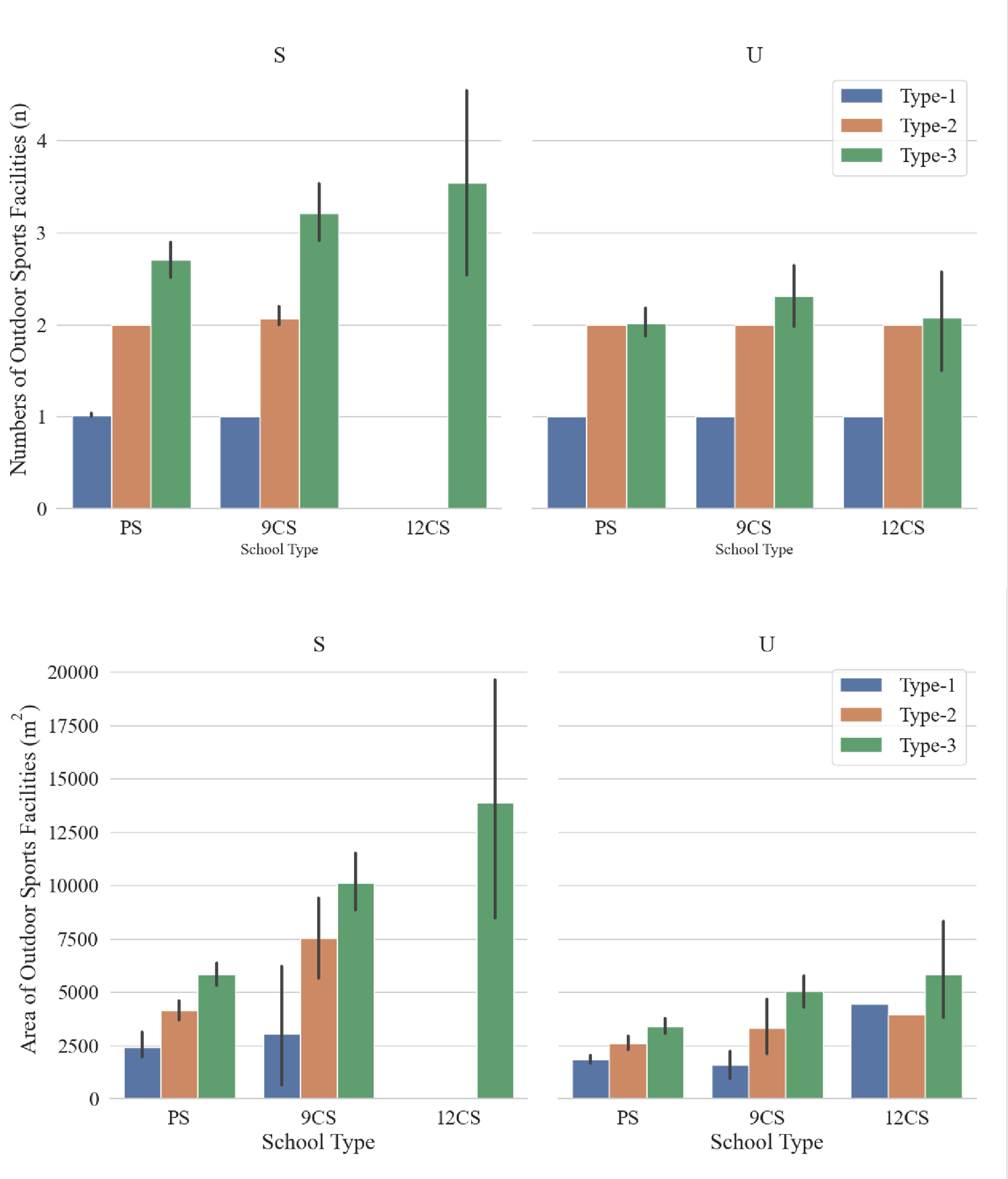

Supplement: S3 Fig — (TIF) [file pone.0267009.s003.tif]
