# Supplementary material for: An unsupervised machine learning approach to evaluate sports facilities condition in primary school
Source: PLoS One. 2022 Apr 20;17(4):e0267009. doi: 10.1371/journal.pone.0267009 (PMC9020747; doi:10.1371/journal.pone.0267009)
Supplement: S1 Dataset — (PDF) [file pone.0267009.s004.pdf]

Samples	Location Type	School Catergory	TT	TA	Average TA	IT	IA	Average IA	OT	OA	Average OA	Location
1	S	9CS	2	3398	1699	0	0	0	2	3398	1699	113.129
2	S	PS	1	501.5	501.5	0	0	0	1	501.5	501.5	42.011
3	S	PS	4	6113	1528.25	1	608	608	3	5505	1835	48.807
4	S	PS	1	360	360	0	0	0	1	360	360	38.739
5	S	PS	2	5897	2948.5	0	0	0	2	5897	2948.5	18.998
6	S	PS	2	8014	4007	0	0	0	2	8014	4007	32.204
7	S	9CS	3	5180	1726.67	1	864	864	2	4316	2158	24.738
8	S	PS	1	6100	6100	0	0	0	1	6100	6100	20.66
9	S	12CS	2	5919	2959.5	1	1050	1050	1	4869	4869	18.937
10	S	PS	2	455	227.5	0	0	0	2	455	227.5	34.66
11	S	PS	4	4770	1192.5	1	910	910	3	3860	1286.67	38.557
12	S	12CS	4	10662	2665.5	0	0	0	4	10662	2665.5	43.967
13	S	PS	1	3080	3080	0	0	0	1	3080	3080	21.678
14	S	PS	1	450	450	0	0	0	1	450	450	30.038
15	U	PS	3	2389.92	796.64	1	42.92	42.92	2	2347	1173.5	9.34
16	S	PS	3	556	185.33	0	0	0	3	556	185.33	82.375
17	S	PS	2	5008	2504	0	0	0	2	5008	2504	21.104
18	U	PS	2	1683	841.5	1	48	48	1	1635	1635	2.307
19	U	12CS	6	4949.5	824.92	2	806	403	4	4143.5	1035.88	8.502
20	U	9CS	5	5117.7	1023.54	1	75	75	4	5042.7	1260.67	8.493
21	U	PS	4	8447	2111.75	2	1102	551	2	7345	3672.5	10.564
22	S	PS	1	2466	2466	1	2466	2466	0	0	0	19.025
23	U	PS	1	1620	1620	0	0	0	1	1620	1620	8.645
24	U	9CS	2	8746	4373	1	665	665	1	8081	8081	11.977
25	S	PS	1	800	800	0	0	0	1	800	800	19.447
26	U	9CS	2	9733.64	4866.82	1	933.64	933.64	1	8800	8800	9.512
27	U	PS	3	5493	1831	2	793	396.5	1	4700	4700	11.881
28	S	PS	4	8089	2022.25	1	738	738	3	7351	2450.33	16.449
29	U	PS	3	8399.08	2799.69	1	871.08	871.08	2	7528	3764	9.269
30	S	PS	2	2121	1060.5	0	0	0	2	2121	1060.5	37.96
31	U	PS	1	1730	1730	0	0	0	1	1730	1730	5.348
32	S	PS	1	945	945	0	0	0	1	945	945	37.639
33	S	PS	2	4888	2444	1	448	448	1	4440	4440	36.114
34	U	PS	2	4850	2425	1	608	608	1	4242	4242	13.767
35	S	PS	2	3568.03	1784.02	1	368.03	368.03	1	3200	3200	15.182
36	S	PS	3	9930.84	3310.28	1	658.84	658.84	2	9272	4636	25.332
37	S	PS	7	19671	2810.14	3	1538	512.67	4	18133	4533.25	16.606
38	S	PS	3	5775	1925	0	0	0	3	5775	1925	23.944
39	U	PS	6	6769.6	1128.27	3	1014.6	338.2	3	5755	1918.33	5.962
40	U	PS	3	1793.64	597.88	1	297.5	297.5	2	1496.14	748.07	2.726
41	U	PS	2	2946	1473	1	221	221	1	2725	2725	13.383
42	U	PS	2	1290	645	0	0	0	2	1290	645	2.708
43	U	PS	2	2408	1204	0	0	0	2	2408	1204	7.732
44	S	PS	6	8859	1476.5	1	842	842	5	8017	1603.4	34.342
45	U	PS	1	1200	1200	0	0	0	1	1200	1200	6.338
46	U	9CS	1	3568.99	3568.99	0	0	0	1	3568.99	3568.99	6.45
47	U	PS	2	1176.54	588.27	1	123.54	123.54	1	1053	1053	4.78
48	U	PS	2	671	335.5	0	0	0	2	671	335.5	2.94
49	U	PS	2	4190	2095	0	0	0	2	4190	2095	8.621
50	U	PS	2	3760	1880	0	0	0	2	3760	1880	4.805
51	S	PS	3	7240	2413.33	0	0	0	3	7240	2413.33	19.844
52	U	PS	2	1216	608	0	0	0	2	1216	608	3.538
53	S	PS	3	4427	1475.67	1	340	340	2	4087	2043.5	47.417
54	S	PS	1	672	672	0	0	0	1	672	672	8.7498
55	U	PS	2	6656.75	3328.38	1	843.75	843.75	1	5813	5813	8.484
56	U	PS	2	2182	1091	0	0	0	2	2182	1091	12.939
57	U	PS	1	462.4	462.4	0	0	0	1	462.4	462.4	2.498
58	U	PS	1	2352.26	2352.26	0	0	0	1	2352.26	2352.26	10.586
59	S	PS	5	14938	2987.6	1	408	408	4	14530	3632.5	31.737
60	S	9CS	1	5694	5694	0	0	0	1	5694	5694	19.473
61	U	PS	2	1646.25	823.12	0	0	0	2	1646.25	823.12	3.445
62	U	9CS	3	3264	1088	2	1440	720	1	1824	1824	11.979
63	S	PS	1	560	560	0	0	0	1	560	560	22.665
64	S	PS	5	12603	2520.6	2	923	461.5	3	11680	3893.33	27.326
65	U	PS	3	928.8	309.6	1	106	106	2	822.8	411.4	2.724
66	U	PS	4	9029	2257.25	1	600	600	3	8429	2809.67	8.043
67	U	9CS	4	3629	907.25	2	924	462	2	2705	1352.5	5.937
68	U	PS	1	1000	1000	0	0	0	1	1000	1000	1.894
69	S	PS	6	6584.28	1097.38	2	981	490.5	4	5603.28	1400.82	22.384
70	U	PS	1	2100	2100	0	0	0	1	2100	2100	6.103
71	U	PS	1	2450	2450	0	0	0	1	2450	2450	9.846

Samples	Location Type	School Catergory	TT	TA	Average TA	IT	IA	Average IA	OT	OA	Average OA	Location
72	U	PS	1	1815.45	1815.45	0	0	0	1	1815.45	1815.45	4.071
73	U	PS	1	1464.48	1464.48	0	0	0	1	1464.48	1464.48	6.729
74	S	9CS	2	10426	5213	0	0	0	2	10426	5213	25.069
75	U	9CS	2	1808	904	0	0	0	2	1808	904	10.919
76	U	PS	2	3668	1834	0	0	0	2	3668	1834	12.741
77	U	PS	2	3060	1530	1	660	660	1	2400	2400	5.794
78	U	9CS	5	7418.74	1483.75	0	0	0	5	7418.74	1483.75	9.284
79	S	PS	2	3252	1626	0	0	0	2	3252	1626	20.374
80	U	PS	2	2751.62	1375.81	0	0	0	2	2751.62	1375.81	9.022
81	U	PS	4	1984	496	0	0	0	4	1984	496	4.749
82	S	PS	3	13170	4390	1	750	750	2	12420	6210	16.324
83	S	PS	5	5281	1056.2	1	46	46	4	5235	1308.75	43.491
84	U	PS	1	1220	1220	0	0	0	1	1220	1220	8.963
85	U	12CS	2	2147	1073.5	1	585	585	1	1562	1562	3.785
86	S	PS	2	2720	1360	0	0	0	2	2720	1360	21.321
87	U	PS	1	2200	2200	0	0	0	1	2200	2200	12.27
88	U	PS	2	5122	2561	0	0	0	2	5122	2561	10.332
89	U	PS	1	3659	3659	0	0	0	1	3659	3659	6.221
90	U	PS	2	2516	1258	0	0	0	2	2516	1258	11.713
91	U	PS	1	2065.5	2065.5	0	0	0	1	2065.5	2065.5	9.436
92	S	9CS	4	9124	2281	1	100	100	3	9024	3008	33.453
93	S	PS	1	4190	4190	0	0	0	1	4190	4190	19.246
94	U	9CS	5	6389	1277.8	2	1406	703	3	4983	1661	4.353
95	U	PS	6	10558	1759.67	2	911	455.5	4	9647	2411.75	11.578
96	S	PS	1	1750	1750	0	0	0	1	1750	1750	27.888
97	U	PS	3	2430	810	0	0	0	3	2430	810	6.984
98	U	9CS	3	5183.39	1727.8	1	84.68	84.68	2	5098.71	2549.36	4.936
99	U	PS	2	3841	1920.5	0	0	0	2	3841	1920.5	5.247
100	U	PS	1	1542	1542	0	0	0	1	1542	1542	4.875
101	S	PS	2	3487	1743.5	0	0	0	2	3487	1743.5	48.25
102	S	PS	2	6608	3304	0	0	0	2	6608	3304	28.042
103	U	PS	2	1268.55	634.27	1	268.55	268.55	1	1000	1000	4.649
104	U	PS	6	7489.38	1248.23	2	606	303	4	6883.38	1720.85	12.212
105	U	PS	1	1410	1410	0	0	0	1	1410	1410	10.143
106	U	PS	1	5394	5394	0	0	0	1	5394	5394	8.443
107	U	12CS	2	3948	1974	0	0	0	2	3948	1974	5.133
108	U	PS	1	375	375	0	0	0	1	375	375	3.546
109	U	PS	1	1185.52	1185.52	0	0	0	1	1185.52	1185.52	4.465
110	S	PS	3	5566.7	1855.57	1	372.5	372.5	2	5194.2	2597.1	20.359
111	S	PS	3	7550	2516.67	1	990	990	2	6560	3280	31.859
112	U	12CS	1	118.8	118.8	1	118.8	118.8	0	0	0	10.345
113	U	9CS	3	6540.03	2180.01	1	300.03	300.03	2	6240	3120	6.796
114	U	PS	1	1026	1026	0	0	0	1	1026	1026	5.662
115	U	PS	1	1200.15	1200.15	0	0	0	1	1200.15	1200.15	4.474
116	S	PS	3	6316	2105.33	1	936	936	2	5380	2690	36.006
117	S	PS	2	4298	2149	0	0	0	2	4298	2149	33.341
118	U	PS	2	3197.1	1598.55	0	0	0	2	3197.1	1598.55	9.734
119	S	9CS	3	8716	2905.33	1	700	700	2	8016	4008	17.279
120	U	PS	2	2903	1451.5	1	195	195	1	2708	2708	12.713
121	U	9CS	3	7557	2519	1	800	800	2	6757	3378.5	6.49
122	U	9CS	3	8530	2843.33	1	680	680	2	7850	3925	11.689
123	S	PS	2	7470	3735	0	0	0	2	7470	3735	17.507
124	U	PS	1	2432	2432	0	0	0	1	2432	2432	11.787
125	S	PS	2	3308	1654	0	0	0	2	3308	1654	21.014
126	U	PS	2	3080	1540	0	0	0	2	3080	1540	12.307
127	U	PS	2	940	470	1	120	120	1	820	820	2.37
128	U	PS	3	3988	1329.33	0	0	0	3	3988	1329.33	11.624
129	U	PS	1	3100	3100	0	0	0	1	3100	3100	7.469
130	U	PS	2	3122	1561	1	396	396	1	2726	2726	14.028
131	S	PS	1	2400	2400	0	0	0	1	2400	2400	16.317
132	U	PS	1	2855	2855	0	0	0	1	2855	2855	5.67
133	S	9CS	7	14828.75	2118.39	0	0	0	7	14828.75	2118.39	67.454
134	U	PS	4	5128	1282	1	360	360	3	4768	1589.33	10.327
135	S	9CS	3	5836	1945.33	2	2200	1100	1	3636	3636	19.609
136	S	9CS	2	5744	2872	0	0	0	2	5744	2872	51.235
137	S	9CS	6	13726	2287.67	1	300	300	5	13426	2685.2	30.938
138	U	9CS	4	5613	1403.25	2	1273	636.5	2	4340	2170	5.425
139	U	PS	2	2241	1120.5	1	541	541	1	1700	1700	3.734
140	U	9CS	1	1400	1400	0	0	0	1	1400	1400	10.145
141	S	9CS	3	7654.4	2551.47	1	862.4	862.4	2	6792	3396	33.309
142	U	PS	1	1685.6	1685.6	0	0	0	1	1685.6	1685.6	4.124

Samples	Location Type	School Catergory	TT	TA	Average TA	IT	IA	Average IA	OT	OA	Average OA	Location
143	S	PS	1	3525	3525	0	0	0	1	3525	3525	39.237
144	S	PS	5	12875	2575	1	330	330	4	12545	3136.25	37.24
145	U	PS	1	6212.2	6212.2	0	0	0	1	6212.2	6212.2	2.826
146	U	PS	1	1811.4	1811.4	0	0	0	1	1811.4	1811.4	5.146
147	S	9CS	6	9943.5	1657.25	3	1815.5	605.17	3	8128	2709.33	17.452
148	U	PS	1	3509	3509	0	0	0	1	3509	3509	5.242
149	S	9CS	4	12534	3133.5	2	1034	517	2	11500	5750	23.982
150	U	PS	1	1462	1462	0	0	0	1	1462	1462	4.877
151	S	PS	1	1600	1600	0	0	0	1	1600	1600	28.294
152	U	PS	1	2450	2450	0	0	0	1	2450	2450	2.971
153	U	PS	2	686	343	0	0	0	2	686	343	2.503
154	S	PS	3	9435	3145	1	111	111	2	9324	4662	26.564
155	U	9CS	5	3204.43	640.89	1	74.75	74.75	4	3129.68	782.42	9.976
156	U	9CS	2	6388	3194	1	1113	1113	1	5275	5275	4.069
157	U	PS	3	3144	1048	1	154	154	2	2990	1495	11.851
158	U	PS	2	4556	2278	0	0	0	2	4556	2278	10.98
159	U	9CS	1	1170	1170	0	0	0	1	1170	1170	9.141
160	S	PS	1	1216	1216	0	0	0	1	1216	1216	20.184
161	U	PS	2	3865	1932.5	1	676	676	1	3189	3189	9.293
162	U	PS	1	2700	2700	0	0	0	1	2700	2700	13.178
163	S	PS	2	1634	817	0	0	0	2	1634	817	21.652
164	S	PS	1	1364	1364	0	0	0	1	1364	1364	16.963
165	U	PS	1	2690	2690	0	0	0	1	2690	2690	11.404
166	U	PS	2	1646	823	1	396	396	1	1250	1250	11.756
167	U	PS	2	587	293.5	0	0	0	2	587	293.5	4.207
168	U	PS	2	1310.4	655.2	0	0	0	2	1310.4	655.2	4.945
169	U	PS	2	764	382	1	404	404	1	360	360	11.856
170	U	9CS	1	3254	3254	0	0	0	1	3254	3254	9.736
171	S	PS	3	8563	2854.33	1	595	595	2	7968	3984	28.423
172	U	PS	2	2750	1375	0	0	0	2	2750	1375	8.09
173	U	PS	1	247.5	247.5	1	247.5	247.5	0	0	0	0.926
174	S	9CS	5	4319	863.8	3	1637	545.67	2	2682	1341	30.891
175	U	PS	2	2230	1115	0	0	0	2	2230	1115	10.043
176	S	9CS	8	10132.38	1266.55	4	1961.63	490.41	4	8170.75	2042.69	35.366
177	U	PS	2	3164	1582	0	0	0	2	3164	1582	11.215
178	S	PS	1	2500	2500	0	0	0	1	2500	2500	15.069
179	U	PS	1	1925	1925	0	0	0	1	1925	1925	5.264
180	U	PS	2	3046.23	1523.12	0	0	0	2	3046.23	1523.12	7.415
181	S	PS	1	736	736	0	0	0	1	736	736	27.999
182	U	PS	3	3371.94	1123.98	0	0	0	3	3371.94	1123.98	9.857
183	S	PS	4	7278	1819.5	0	0	0	4	7278	1819.5	49.682
184	U	PS	3	3780	1260	1	700	700	2	3080	1540	13.337
185	U	PS	1	1320	1320	0	0	0	1	1320	1320	8.417
186	S	9CS	8	33090.12	4136.27	1	893.56	893.56	7	32196.56	4599.51	53.274
187	S	PS	2	7450	3725	0	0	0	2	7450	3725	15.365
188	U	PS	3	2157	719	1	299	299	2	1858	929	8.207
189	U	9CS	3	3456	1152	1	159	159	2	3297	1648.5	5.388
190	U	PS	2	8290	4145	1	680	680	1	7610	7610	13.331
191	S	PS	3	8340	2780	1	805	805	2	7535	3767.5	28.159
192	S	PS	1	3628.8	3628.8	0	0	0	1	3628.8	3628.8	25.51
193	S	9CS	3	7573	2524.33	1	640	640	2	6933	3466.5	31.841
194	S	PS	3	7681	2560.33	1	847	847	2	6834	3417	46.372
195	S	PS	1	4751	4751	0	0	0	1	4751	4751	59.557
196	S	PS	1	850	850	0	0	0	1	850	850	17.862
197	S	PS	3	8217	2739	1	875	875	2	7342	3671	31.513
198	U	PS	4	1679.17	419.79	2	505.57	252.78	2	1173.6	586.8	2.388
199	S	PS	3	3300	1100	1	720	720	2	2580	1290	17.242
200	U	PS	1	1349	1349	0	0	0	1	1349	1349	6.932
201	U	PS	1	2300.32	2300.32	0	0	0	1	2300.32	2300.32	6.833
202	U	PS	2	2367	1183.5	0	0	0	2	2367	1183.5	11.414
203	U	9CS	3	9282	3094	1	910	910	2	8372	4186	10.123
204	U	PS	2	5321	2660.5	0	0	0	2	5321	2660.5	8.711
205	U	PS	3	881.09	293.7	1	107.67	107.67	2	773.42	386.71	3.029
206	U	PS	4	830.29	207.57	2	65.13	32.56	2	765.16	382.58	4.28
207	S	PS	2	7149	3574.5	0	0	0	2	7149	3574.5	29.93
208	S	PS	1	750	750	0	0	0	1	750	750	22.442
209	S	12CS	7	15389	2198.43	2	806	403	5	14583	2916.6	18.029
210	S	PS	3	5028	1676	1	360	360	2	4668	2334	43.511
211	U	PS	1	2617	2617	0	0	0	1	2617	2617	10.189
212	S	PS	1	1887	1887	0	0	0	1	1887	1887	23.018
213	U	PS	3	1700	566.67	0	0	0	3	1700	566.67	7.552

Samples	Location Type	School Catergory	TT	TA	Average TA	IT	IA	Average IA	OT	OA	Average OA	Location
214	U	PS	2	1155.9	577.95	0	0	0	2	1155.9	577.95	3.146
215	S	PS	7	9388.9	1341.27	1	205	205	6	9183.9	1530.65	43.188
216	U	PS	3	5565	1855	1	507	507	2	5058	2529	12.964
217	S	PS	2	6777	3388.5	1	777	777	1	6000	6000	15.4
218	U	9CS	5	7945.5	1589.1	3	841.5	280.5	2	7104	3552	12.666
219	U	9CS	2	977.1	488.55	1	127.1	127.1	1	850	850	4.807
220	U	PS	3	2392.06	797.35	1	96.76	96.76	2	2295.3	1147.65	5.732
221	U	PS	3	1750	583.33	2	292	146	1	1458	1458	7.126
222	S	PS	2	6016	3008	0	0	0	2	6016	3008	80.336
223	S	PS	2	1715	857.5	0	0	0	2	1715	857.5	79.062
224	U	PS	1	994.4	994.4	0	0	0	1	994.4	994.4	5.911
225	U	9CS	2	2932	1466	0	0	0	2	2932	1466	8.887
226	S	PS	4	7737	1934.25	1	720	720	3	7017	2339	26.432
227	U	9CS	4	8246	2061.5	0	0	0	4	8246	2061.5	8.126
228	U	PS	3	3967.8	1322.6	1	448.8	448.8	2	3519	1759.5	8.586
229	U	9CS	2	4134	2067	0	0	0	2	4134	2067	5.94
230	U	PS	1	3808	3808	0	0	0	1	3808	3808	10.304
231	U	PS	3	3688.65	1229.55	1	203.85	203.85	2	3484.8	1742.4	7.191
232	U	PS	1	1025	1025	0	0	0	1	1025	1025	6.309
233	S	9CS	4	3712	928	0	0	0	4	3712	928	57.547
234	S	PS	2	813	406.5	0	0	0	2	813	406.5	35.883
235	S	PS	2	4911	2455.5	0	0	0	2	4911	2455.5	27.995
236	U	12CS	4	2531	632.75	2	551	275.5	2	1980	990	3.946
237	S	PS	2	3568	1784	0	0	0	2	3568	1784	44.018
238	U	PS	3	1838	612.67	1	400	400	2	1438	719	9.839
239	U	PS	2	4580	2290	0	0	0	2	4580	2290	11.656
240	S	PS	1	1216	1216	0	0	0	1	1216	1216	23.164
241	U	PS	1	1500	1500	0	0	0	1	1500	1500	7.718
242	U	PS	4	6204	1551	1	680	680	3	5524	1841.33	9.318
243	U	PS	2	3523	1761.5	0	0	0	2	3523	1761.5	14.357
244	U	PS	1	530.79	530.79	0	0	0	1	530.79	530.79	3.56
245	U	PS	4	2385	596.25	1	166	166	3	2219	739.67	10.089
246	S	PS	1	608	608	0	0	0	1	608	608	29.412
247	U	PS	1	406.12	406.12	0	0	0	1	406.12	406.12	2.175
248	S	PS	2	5012	2506	1	440	440	1	4572	4572	16.494
249	U	PS	3	3330	1110	1	450	450	2	2880	1440	9.821
250	S	PS	4	7765	1941.25	1	1200	1200	3	6565	2188.33	15.635
251	U	PS	1	1690.7	1690.7	0	0	0	1	1690.7	1690.7	4.95
252	U	9CS	4	2669.37	667.34	2	560.12	280.06	2	2109.25	1054.62	1.559
253	S	PS	2	1057.63	528.82	0	0	0	2	1057.63	528.82	15.325
254	S	PS	2	3256	1628	0	0	0	2	3256	1628	17.597
255	U	PS	3	8827	2942.33	0	0	0	3	8827	2942.33	12.207
256	S	PS	1	306	306	0	0	0	1	306	306	33.818
257	U	PS	3	942	314	0	0	0	3	942	314	9.521
258	S	PS	1	1216	1216	0	0	0	1	1216	1216	33.45
259	S	PS	1	1216	1216	0	0	0	1	1216	1216	33.583
260	S	PS	3	8016	2672	1	816	816	2	7200	3600	21.028
261	S	PS	1	375	375	0	0	0	1	375	375	24.668
262	S	PS	1	1540	1540	0	0	0	1	1540	1540	26.432
263	S	PS	2	7202	3601	1	792	792	1	6410	6410	16.611
264	S	PS	4	7200	1800	1	864	864	3	6336	2112	19.9
265	U	PS	3	1190	396.67	2	90	45	1	1100	1100	8.944
266	U	PS	1	608	608	0	0	0	1	608	608	4.658
267	U	PS	3	6636	2212	1	875	875	2	5761	2880.5	12.321
268	U	PS	2	1186.86	593.43	0	0	0	2	1186.86	593.43	4.183
269	U	PS	2	2120	1060	1	500	500	1	1620	1620	8.767
270	U	PS	2	2953	1476.5	0	0	0	2	2953	1476.5	8.842
271	U	PS	1	2450	2450	0	0	0	1	2450	2450	7.23
272	U	PS	4	4910	1227.5	3	1532	510.67	1	3378	3378	3.413
273	U	12CS	7	8931	1275.86	5	2929	585.8	2	6002	3001	11.145
274	U	PS	2	2049	1024.5	0	0	0	2	2049	1024.5	8.891
275	S	PS	2	2400	1200	0	0	0	2	2400	1200	16.685
276	S	PS	2	5253	2626.5	0	0	0	2	5253	2626.5	56.767
277	U	9CS	2	1933	966.5	1	165	165	1	1768	1768	5.509
278	U	PS	3	5074	1691.33	1	600	600	2	4474	2237	9.33
279	S	12CS	3	5023	1674.33	1	1280	1280	2	3743	1871.5	19.003
280	S	9CS	5	6722.1	1344.42	1	406	406	4	6316.1	1579.03	17.257
281	U	PS	2	1896	948	0	0	0	2	1896	948	9.21
282	U	PS	1	852	852	0	0	0	1	852	852	1.591
283	S	PS	5	6519.2	1303.84	2	259.2	129.6	3	6260	2086.67	27.621
284	S	PS	1	700	700	0	0	0	1	700	700	24.702

Samples	Location Type	School Catergory	TT	TA	Average TA	IT	IA	Average IA	OT	OA	Average OA	Location
285	U	PS	1	6000	6000	0	0	0	1	6000	6000	12.582
286	S	PS	2	7361	3680.5	0	0	0	2	7361	3680.5	26.413
287	S	9CS	3	5594	1864.67	1	760	760	2	4834	2417	27.432
288	S	9CS	5	7520	1504	0	0	0	5	7520	1504	27.143
289	U	9CS	3	6552	2184	0	0	0	3	6552	2184	10.696
290	S	PS	1	495	495	0	0	0	1	495	495	16.205
291	S	PS	3	5357	1785.67	1	772	772	2	4585	2292.5	57.496
292	S	PS	3	5772	1924	1	608	608	2	5164	2582	23.189
293	S	PS	1	2200	2200	0	0	0	1	2200	2200	17.128
294	S	PS	2	1393	696.5	0	0	0	2	1393	696.5	28.241
295	U	12CS	4	6792	1698	3	1876	625.33	1	4916	4916	9.678
296	U	PS	3	5636.5	1878.83	1	559.5	559.5	2	5077	2538.5	12.204
297	S	PS	1	2800	2800	0	0	0	1	2800	2800	73.366
298	S	PS	3	8654	2884.67	1	1030	1030	2	7624	3812	32.211
299	U	PS	1	1739	1739	0	0	0	1	1739	1739	7.218
300	U	PS	2	822.5	411.25	0	0	0	2	822.5	411.25	7.087
301	S	PS	2	3100	1550	0	0	0	2	3100	1550	24.771
302	U	PS	1	2014.92	2014.92	0	0	0	1	2014.92	2014.92	11.517
303	S	PS	3	5446	1815.33	1	230	230	2	5216	2608	34.385
304	S	PS	2	505.1	252.55	0	0	0	2	505.1	252.55	19.431
305	U	PS	1	1000	1000	0	0	0	1	1000	1000	10.486
306	S	PS	2	8250	4125	0	0	0	2	8250	4125	44.759
307	U	PS	1	4400	4400	0	0	0	1	4400	4400	10.042
308	S	PS	2	5305.6	2652.8	0	0	0	2	5305.6	2652.8	53.8
309	S	PS	1	2800	2800	0	0	0	1	2800	2800	35.807
310	S	PS	2	1254	627	0	0	0	2	1254	627	33.551
311	S	PS	2	3600	1800	0	0	0	2	3600	1800	39.499
312	S	PS	3	1944	648	1	116	116	2	1828	914	20.577
313	U	PS	3	6978	2326	1	1815	1815	2	5163	2581.5	8.477
314	U	PS	1	1824	1824	0	0	0	1	1824	1824	8.814
315	U	PS	3	2204	734.67	0	0	0	3	2204	734.67	8.717
316	U	PS	2	2306.9	1153.45	0	0	0	2	2306.9	1153.45	9.511
317	U	PS	3	7291	2430.33	1	326	326	2	6965	3482.5	11.605
318	U	PS	2	862	431	1	216	216	1	646	646	7.778
319	U	PS	1	928	928	0	0	0	1	928	928	10.786
320	U	9CS	3	6242	2080.67	1	650	650	2	5592	2796	11.999
321	U	PS	2	5949	2974.5	1	420	420	1	5529	5529	11.952
322	S	12CS	9	28303	3144.78	5	4956	991.2	4	23347	5836.75	18.619
323	S	PS	1	5080	5080	0	0	0	1	5080	5080	36.143
324	U	9CS	5	6719	1343.8	3	1488	496	2	5231	2615.5	3.602
325	U	PS	1	588	588	0	0	0	1	588	588	5.943
326	U	PS	1	1500	1500	0	0	0	1	1500	1500	4.724
327	U	PS	2	1877.69	938.85	1	263.73	263.73	1	1613.96	1613.96	6.211
328	U	PS	4	4271	1067.75	2	625	312.5	2	3646	1823	6.489
329	U	PS	1	1390.6	1390.6	0	0	0	1	1390.6	1390.6	1.378
330	S	9CS	2	2993.53	1496.77	0	0	0	2	2993.53	1496.77	15.792
331	S	PS	1	937	937	0	0	0	1	937	937	16.006
332	U	PS	3	1251	417	1	411	411	2	840	420	7.664
333	S	9CS	4	12185.68	3046.42	1	1200	1200	3	10985.68	3661.89	35.703
334	U	PS	1	881.5	881.5	0	0	0	1	881.5	881.5	6.74
335	U	12CS	5	10209	2041.8	1	2070	2070	4	8139	2034.75	8.141
336	U	PS	3	4475	1491.67	1	314	314	2	4161	2080.5	11.994
337	S	9CS	4	18087	4521.75	2	1199	599.5	2	16888	8444	32.145
338	U	PS	1	3400	3400	0	0	0	1	3400	3400	6.99
339	S	PS	3	1745	581.67	1	115	115	2	1630	815	54.986
340	U	PS	3	4825.84	1608.61	1	392.04	392.04	2	4433.8	2216.9	10.278
341	U	PS	3	2610.7	870.23	1	161.6	161.6	2	2449.1	1224.55	7.766
342	U	PS	2	838.51	419.25	0	0	0	2	838.51	419.25	3.396
343	S	9CS	3	7828	2609.33	0	0	0	3	7828	2609.33	15.31
344	U	9CS	3	4530	1510	1	150	150	2	4380	2190	8.312
345	U	PS	3	4040	1346.67	1	520	520	2	3520	1760	13.177
346	S	PS	3	5642	1880.67	1	252	252	2	5390	2695	17.168
347	S	9CS	3	17300	5766.67	0	0	0	3	17300	5766.67	48.037
348	S	PS	1	5839	5839	0	0	0	1	5839	5839	42.889
349	S	PS	2	3408	1704	0	0	0	2	3408	1704	19.25
350	U	PS	2	775.65	387.82	1	313.65	313.65	1	462	462	4.288
351	U	PS	3	2030	676.67	1	286	286	2	1744	872	8.899
352	U	PS	3	2403	801	0	0	0	3	2403	801	10.349
353	S	PS	5	5951.82	1190.36	2	678.2	339.1	3	5273.62	1757.87	18.799
354	S	9CS	2	9930	4965	0	0	0	2	9930	4965	36.186
355	S	12CS	11	39984	3634.91	5	2896	579.2	6	37088	6181.33	30.841

Samples	Location Type	School Catergory	TT	TA	Average TA	IT	IA	Average IA	OT	OA	Average OA	Location
356	U	PS	4	2945	736.25	1	400	400	3	2545	848.33	12.67
357	U	9CS	3	3566	1188.67	2	706	353	1	2860	2860	7.823
358	S	9CS	6	17757.67	2959.61	2	457.06	228.53	4	17300.61	4325.15	45.613
359	S	PS	2	97	48.5	1	67	67	1	30	30	52.28
360	S	PS	6	7860.2	1310.03	1	1208	1208	5	6652.2	1330.44	33.802
361	U	9CS	4	6342.1	1585.53	1	510	510	3	5832.1	1944.03	7.941
362	U	PS	3	920.42	306.81	0	0	0	3	920.42	306.81	6.921
363	U	PS	1	3528	3528	0	0	0	1	3528	3528	7.7
364	U	PS	1	1850	1850	0	0	0	1	1850	1850	12.546
365	S	PS	3	14607	4869	1	208	208	2	14399	7199.5	23.547
366	S	PS	4	5011.2	1252.8	1	367.2	367.2	3	4644	1548	28
367	S	PS	3	3765.2	1255.07	1	77.2	77.2	2	3688	1844	51.153
368	S	PS	2	4948	2474	0	0	0	2	4948	2474	25.598
369	S	PS	2	5716	2858	0	0	0	2	5716	2858	40.068
370	U	PS	3	1221	407	1	432	432	2	789	394.5	9.147
371	S	9CS	4	7226	1806.5	1	387	387	3	6839	2279.67	51.176
372	U	9CS	4	9312.6	2328.15	2	1585.9	792.95	2	7726.7	3863.35	11.392
373	U	PS	3	4840	1613.33	1	300	300	2	4540	2270	13.276
374	S	PS	3	4359	1453	1	579	579	2	3780	1890	15.284
375	S	PS	1	336	336	0	0	0	1	336	336	34.235
376	S	PS	1	1216	1216	0	0	0	1	1216	1216	49.335
377	U	PS	2	2928	1464	0	0	0	2	2928	1464	8.234
378	U	PS	1	1530	1530	0	0	0	1	1530	1530	4.704
379	U	PS	1	1009	1009	0	0	0	1	1009	1009	2.508
380	U	PS	1	1485	1485	0	0	0	1	1485	1485	3.855
381	S	PS	4	7049.2	1762.3	1	155.2	155.2	3	6894	2298	19.636
382	S	PS	2	6135.56	3067.78	0	0	0	2	6135.56	3067.78	17.93
383	S	PS	1	4600	4600	0	0	0	1	4600	4600	23.024
384	U	PS	1	1029	1029	0	0	0	1	1029	1029	2.75
385	U	PS	3	8683	2894.33	1	782	782	2	7901	3950.5	10.559
386	U	PS	1	2998	2998	0	0	0	1	2998	2998	6.668
387	S	PS	1	150	150	0	0	0	1	150	150	42.736
388	S	9CS	5	22684.8	4536.96	2	974.6	487.3	3	21710.2	7236.73	26.876
389	S	9CS	1	1216	1216	0	0	0	1	1216	1216	39.965
390	S	PS	4	11419	2854.75	2	972	486	2	10447	5223.5	23.167
391	S	PS	2	6324	3162	0	0	0	2	6324	3162	50.109
392	S	9CS	3	10198	3399.33	1	578	578	2	9620	4810	17.451
393	S	PS	2	591	295.5	0	0	0	2	591	295.5	20.328
394	S	PS	2	5260	2630	0	0	0	2	5260	2630	36.623
395	U	PS	1	1280	1280	0	0	0	1	1280	1280	10719
396	S	PS	2	4852	2426	0	0	0	2	4852	2426	42.162
397	U	PS	1	1575	1575	0	0	0	1	1575	1575	4.968
398	S	12CS	4	6427	1606.75	2	2740	1370	2	3687	1843.5	19.188
399	U	9CS	1	777	777	0	0	0	1	777	777	4.905
400	S	PS	2	6188.8	3094.4	0	0	0	2	6188.8	3094.4	42.848
401	U	PS	1	1216	1216	0	0	0	1	1216	1216	4.96
402	U	PS	1	673.2	673.2	0	0	0	1	673.2	673.2	4.686
403	S	12CS	8	31455	3931.88	2	2011	1005.5	6	29444	4907.33	18.954
404	S	9CS	3	5640	1880	1	792	792	2	4848	2424	19.036
405	U	PS	1	1200	1200	0	0	0	1	1200	1200	6.238
406	U	9CS	2	2309	1154.5	1	300	300	1	2009	2009	1.195
407	U	PS	2	1168.08	584.04	1	288	288	1	880.08	880.08	12.311
408	U	PS	3	1688.5	562.83	1	175	175	2	1513.5	756.75	1.911
409	S	PS	4	1734	433.5	2	710	355	2	1024	512	44.513
410	U	PS	1	572	572	0	0	0	1	572	572	3.533
411	S	PS	1	5000	5000	0	0	0	1	5000	5000	38.646
412	U	PS	1	1455.3	1455.3	0	0	0	1	1455.3	1455.3	6.16
413	U	PS	4	2330	582.5	2	222	111	2	2108	1054	6.769
414	U	9CS	1	920	920	0	0	0	1	920	920	5.233
415	S	PS	2	4212	2106	1	756	756	1	3456	3456	33.389
416	S	PS	1	125	125	0	0	0	1	125	125	52.661
417	S	9CS	3	8149	2716.33	1	875	875	2	7274	3637	16.574
418	U	PS	3	8090	2696.67	1	900	900	2	7190	3595	14.652
419	U	9CS	1	600	600	0	0	0	1	600	600	13.857
420	S	9CS	2	5440	2720	0	0	0	2	5440	2720	61.062
421	U	PS	3	5154	1718	1	906	906	2	4248	2124	12.981
422	S	PS	4	7928	1982	1	320	320	3	7608	2536	33.371
423	S	PS	2	6820.75	3410.38	0	0	0	2	6820.75	3410.38	44.338
424	S	PS	2	2432	1216	0	0	0	2	2432	1216	19.504
425	U	PS	1	1175	1175	0	0	0	1	1175	1175	8.318
426	S	PS	3	9812	3270.67	1	608	608	2	9204	4602	17.715

Samples	Location Type	School Catergory	TT	TA	Average TA	IT	IA	Average IA	OT	OA	Average OA	Location
427	U	PS	3	3596.33	1198.78	1	421.98	421.98	2	3174.35	1587.17	9.264
428	U	PS	1	1785	1785	0	0	0	1	1785	1785	10.187
429	U	PS	5	5868	1173.6	1	400	400	4	5468	1367	11.872
430	S	9CS	5	19115.3	3823.06	0	0	0	5	19115.3	3823.06	55.236
431	U	PS	3	3527.08	1175.69	1	345.6	345.6	2	3181.48	1590.74	7.22
432	U	PS	3	2421.76	807.25	1	375	375	2	2046.76	1023.38	6.523
433	S	PS	2	3408	1704	0	0	0	2	3408	1704	33.799
434	S	PS	2	718	359	0	0	0	2	718	359	46.966
435	U	9CS	1	4416	4416	0	0	0	1	4416	4416	5.533
436	U	9CS	1	825	825	0	0	0	1	825	825	4.556
437	S	PS	5	5192	1038.4	1	364	364	4	4828	1207	48.698
438	U	PS	4	7052.6	1763.15	2	2334.6	1167.3	2	4718	2359	13.39
439	U	PS	2	1066.8	533.4	1	76.8	76.8	1	990	990	4.671
440	U	PS	2	927.99	464	1	216	216	1	711.99	711.99	2.63
441	U	9CS	1	513.5	513.5	0	0	0	1	513.5	513.5	2.283
442	S	PS	2	852.25	426.12	1	252	252	1	600.25	600.25	17.886
443	U	9CS	3	9951	3317	1	855	855	2	9096	4548	10.69
444	U	PS	3	6738.84	2246.28	1	818.84	818.84	2	5920	2960	9.132
445	U	PS	3	1294	431.33	0	0	0	3	1294	431.33	4.263
446	U	PS	3	4943.53	1647.84	1	363.75	363.75	2	4579.78	2289.89	9.089
447	S	9CS	3	8438	2812.67	1	588	588	2	7850	3925	18.234
448	S	PS	3	1590	530	1	162	162	2	1428	714	32.932
449	S	PS	2	4450	2225	0	0	0	2	4450	2225	24.094
450	U	PS	3	4739	1579.67	1	372	372	2	4367	2183.5	13.17
451	S	PS	3	3040	1013.33	2	700	350	1	2340	2340	16.787
452	S	PS	3	1845	615	0	0	0	3	1845	615	58.221
453	U	PS	2	3764	1882	0	0	0	2	3764	1882	11.441
454	U	9CS	4	8404.68	2101.17	0	0	0	4	8404.68	2101.17	6.9
455	U	PS	1	522	522	0	0	0	1	522	522	1.021
456	U	PS	3	1481	493.67	0	0	0	3	1481	493.67	4.83
457	U	PS	1	2414	2414	0	0	0	1	2414	2414	6.258
458	S	PS	1	360	360	0	0	0	1	360	360	24.755
459	U	PS	1	5230	5230	0	0	0	1	5230	5230	14.247
460	S	PS	3	6409	2136.33	1	693	693	2	5716	2858	17.488
461	S	PS	2	5350	2675	1	200	200	1	5150	5150	18.317
462	S	PS	3	3550	1183.33	0	0	0	3	3550	1183.33	39.298
463	U	PS	3	1528.94	509.65	1	366.13	366.13	2	1162.81	581.4	6.085
464	U	PS	2	1197	598.5	1	406	406	1	791	791	5.701
465	U	9CS	2	1850.98	925.49	1	506.98	506.98	1	1344	1344	3.705
466	U	9CS	3	2112.3	704.1	1	283.8	283.8	2	1828.5	914.25	2.362
467	S	PS	3	8374.2	2791.4	1	771.8	771.8	2	7602.4	3801.2	23.633
468	S	PS	1	6070	6070	0	0	0	1	6070	6070	39.174
469	S	9CS	5	10993.5	2198.7	0	0	0	5	10993.5	2198.7	50.342
470	U	PS	1	600	600	0	0	0	1	600	600	1.077
471	U	PS	2	1950	975	0	0	0	2	1950	975	8.929
472	U	9CS	4	2476.94	619.24	1	134.64	134.64	3	2342.3	780.77	10.68
473	S	9CS	2	10802	5401	0	0	0	2	10802	5401	22.965
474	S	9CS	5	10957	2191.4	1	739	739	4	10218	2554.5	50.114
475	U	PS	4	3090.42	772.61	1	185.22	185.22	3	2905.2	968.4	6.94
476	S	PS	1	2400	2400	0	0	0	1	2400	2400	22.061
477	U	PS	3	4143.2	1381.07	1	835.2	835.2	2	3308	1654	14.769
478	U	PS	1	2262	2262	0	0	0	1	2262	2262	5.914
479	U	PS	1	1573	1573	0	0	0	1	1573	1573	3.516
480	U	PS	2	1916	958	0	0	0	2	1916	958	8.616
481	U	PS	2	2258	1129	0	0	0	2	2258	1129	11.328
482	S	PS	2	7016	3508	0	0	0	2	7016	3508	46.222
483	U	PS	4	11881	2970.25	1	820	820	3	11061	3687	10.465
484	U	PS	3	3480	1160	0	0	0	3	3480	1160	7.076
485	U	PS	3	2290.25	763.42	2	290.25	145.12	1	2000	2000	8.079
486	S	PS	2	4740	2370	1	117	117	1	4623	4623	34.963
487	S	PS	1	648	648	0	0	0	1	648	648	25.11
488	S	PS	1	2200	2200	0	0	0	1	2200	2200	17.248
489	U	PS	4	4614	1153.5	1	360	360	3	4254	1418	13.732
490	U	PS	2	1328.67	664.34	1	46.17	46.17	1	1282.5	1282.5	4.071
491	S	9CS	3	9646	3215.33	0	0	0	3	9646	3215.33	36.931
492	S	PS	3	3051	1017	0	0	0	3	3051	1017	39.249
493	S	9CS	3	12154	4051.33	1	745	745	2	11409	5704.5	31.433
494	U	PS	3	4848.09	1616.03	1	216.09	216.09	2	4632	2316	9.488
495	U	PS	2	2466.25	1233.12	1	26.25	26.25	1	2440	2440	7.711
496	U	PS	1	1290	1290	0	0	0	1	1290	1290	3.235
497	U	PS	1	1200	1200	0	0	0	1	1200	1200	6.463

Samples	Location Type	School Catergory	TT	TA	Average TA	IT	IA	Average IA	OT	OA	Average OA	Location
498	S	PS	5	5209	1041.8	1	32	32	4	5177	1294.25	20.06
499	S	PS	3	4978	1659.33	0	0	0	3	4978	1659.33	40.446
500	S	PS	3	2792	930.67	1	256	256	2	2536	1268	16.777
501	S	PS	2	2764	1382	0	0	0	2	2764	1382	15
502	U	PS	5	6427.03	1285.41	1	608	608	4	5819.03	1454.76	5.156
503	U	PS	6	3645	607.5	0	0	0	6	3645	607.5	6.378
504	S	9CS	4	21104	5276	1	1134	1134	3	19970	6656.67	39.687
505	S	PS	6	7034	1172.33	2	456	228	4	6578	1644.5	24.022
506	S	PS	2	2943	1471.5	0	0	0	2	2943	1471.5	32.531
507	S	PS	1	2250	2250	0	0	0	1	2250	2250	27.179
508	S	PS	2	4008	2004	0	0	0	2	4008	2004	26.694
509	S	9CS	5	9559	1911.8	0	0	0	5	9559	1911.8	31.206
510	S	PS	5	7284	1456.8	1	550	550	4	6734	1683.5	16.312
511	U	PS	1	1491	1491	0	0	0	1	1491	1491	4.482
512	S	PS	3	5556	1852	1	300	300	2	5256	2628	16.254
513	S	PS	3	4228	1409.33	0	0	0	3	4228	1409.33	27.473
514	S	PS	2	6308.2	3154.1	1	655.2	655.2	1	5653	5653	17.402
515	S	PS	3	3353.7	1117.9	0	0	0	3	3353.7	1117.9	35.214
516	S	9CS	3	13527	4509	1	1020	1020	2	12507	6253.5	40.51
517	S	9CS	3	16986	5662	1	40	40	2	16946	8473	43.579
518	U	PS	2	5428.6	2714.3	0	0	0	2	5428.6	2714.3	6.354
519	U	9CS	5	1640.87	328.17	0	0	0	5	1640.87	328.17	5.984
520	U	9CS	1	840	840	0	0	0	1	840	840	1.944
521	U	PS	1	1247.8	1247.8	0	0	0	1	1247.8	1247.8	13.966
522	S	PS	3	2723	907.67	1	245	245	2	2478	1239	47.398
523	S	PS	4	7704	1926	1	520	520	3	7184	2394.67	18.388
524	S	12CS	3	15534	5178	1	840	840	2	14694	7347	27.956
525	U	PS	1	550	550	0	0	0	1	550	550	2.262
526	U	PS	1	1216	1216	0	0	0	1	1216	1216	8.722
527	S	PS	1	1125	1125	0	0	0	1	1125	1125	34.284
528	U	PS	3	3621.75	1207.25	1	720	720	2	2901.75	1450.88	7.33
529	U	PS	2	3033.75	1516.88	0	0	0	2	3033.75	1516.88	4.731
530	U	PS	3	4696	1565.33	1	252	252	2	4444	2222	9.28
531	S	PS	3	4734	1578	0	0	0	3	4734	1578	50.62
532	S	PS	2	1502	751	0	0	0	2	1502	751	27.991
533	S	9CS	6	26224	4370.67	1	805	805	5	25419	5083.8	30.587
534	S	PS	4	6233	1558.25	1	704	704	3	5529	1843	30.481
535	U	PS	1	2551.5	2551.5	0	0	0	1	2551.5	2551.5	11.958
536	U	PS	2	1450	725	0	0	0	2	1450	725	2.029
537	S	PS	2	3067.54	1533.77	0	0	0	2	3067.54	1533.77	29.935
538	S	9CS	7	14315	2045	4	1115	278.75	3	13200	4400	33.361
539	S	PS	1	1399.5	1399.5	0	0	0	1	1399.5	1399.5	18.813
540	S	9CS	5	7437.84	1487.57	1	175.1	175.1	4	7262.74	1815.68	41.269
541	S	PS	2	4680	2340	0	0	0	2	4680	2340	38.631
542	U	PS	3	4648.61	1549.54	1	350.61	350.61	2	4298	2149	10.499
543	U	PS	3	4064	1354.67	0	0	0	3	4064	1354.67	10.158
544	U	12CS	3	13272	4424	1	952	952	2	12320	6160	13.857
545	S	PS	1	1083.73	1083.73	0	0	0	1	1083.73	1083.73	20.304
546	S	PS	5	7455	1491	1	752	752	4	6703	1675.75	54.731
547	U	PS	1	600	600	0	0	0	1	600	600	2.274
548	S	PS	2	2837	1418.5	1	140	140	1	2697	2697	25.898
549	U	PS	3	2264.8	754.93	1	135	135	2	2129.8	1064.9	10.152
550	S	9CS	4	6756	1689	2	858	429	2	5898	2949	38.3
551	U	PS	3	1601	533.67	1	242	242	2	1359	679.5	3.204
552	U	PS	1	1074.06	1074.06	0	0	0	1	1074.06	1074.06	10.516
553	U	PS	1	670	670	0	0	0	1	670	670	1.006
554	S	PS	3	5925.6	1975.2	1	345.6	345.6	2	5580	2790	41.04
555	S	9CS	5	13366.7	2673.34	2	1566	783	3	11800.7	3933.57	34.545
556	S	PS	4	5334	1333.5	0	0	0	4	5334	1333.5	48.47
557	U	PS	1	2193.75	2193.75	0	0	0	1	2193.75	2193.75	12.349
558	U	9CS	8	9854.5	1231.81	5	4762.5	952.5	3	5092	1697.33	2.105
559	U	PS	2	4129.5	2064.75	1	412.5	412.5	1	3717	3717	8.131
560	S	PS	1	2191	2191	0	0	0	1	2191	2191	24.957
561	S	PS	4	5594	1398.5	1	611	611	3	4983	1661	24.309
562	S	PS	1	2000	2000	0	0	0	1	2000	2000	25.763
563	U	PS	4	2131	532.75	1	360	360	3	1771	590.33	5.165
564	S	PS	5	5347	1069.4	1	608	608	4	4739	1184.75	52.879
565	S	9CS	3	8579	2859.67	1	700	700	2	7879	3939.5	35.627
566	S	PS	2	4312	2156	0	0	0	2	4312	2156	33.832
567	U	PS	2	1602.5	801.25	1	370	370	1	1232.5	1232.5	1.205
568	U	PS	3	3652.8	1217.6	1	46.8	46.8	2	3606	1803	7.404

Samples	Location Type	School Catergory	TT	TA	Average TA	IT	IA	Average IA	OT	OA	Average OA	Location
569	S	PS	4	3690	922.5	1	332	332	3	3358	1119.33	21.901
570	S	PS	2	1840	920	0	0	0	2	1840	920	38.52
571	S	PS	4	9581.3	2395.32	1	322	322	3	9259.3	3086.43	19.056
572	S	PS	1	4919.7	4919.7	0	0	0	1	4919.7	4919.7	23.123
573	U	PS	2	3339.98	1669.99	0	0	0	2	3339.98	1669.99	5.162
574	S	PS	4	6900	1725	1	341	341	3	6559	2186.33	50.603
575	S	9CS	2	7732.6	3866.3	0	0	0	2	7732.6	3866.3	32.231
576	U	9CS	3	5480.04	1826.68	2	980.04	490.02	1	4500	4500	8.181
577	U	PS	3	2461.25	820.42	1	40	40	2	2421.25	1210.62	10.856
578	S	PS	3	4650	1550	1	430	430	2	4220	2110	32.296
579	S	PS	1	700	700	0	0	0	1	700	700	28.633
580	U	PS	3	1903	634.33	2	883	441.5	1	1020	1020	2.05
581	S	9CS	4	8307	2076.75	1	800	800	3	7507	2502.33	28.813
582	U	PS	1	858	858	0	0	0	1	858	858	3.447
583	U	PS	1	910	910	0	0	0	1	910	910	11.587
584	U	PS	2	2951	1475.5	0	0	0	2	2951	1475.5	5.27
585	S	PS	1	3790	3790	0	0	0	1	3790	3790	38.264
586	S	PS	3	3862	1287.33	1	595	595	2	3267	1633.5	22.431
587	U	12CS	4	5706	1426.5	1	189	189	3	5517	1839	14.688
588	S	PS	1	2720	2720	0	0	0	1	2720	2720	50.928
589	S	9CS	3	3745	1248.33	0	0	0	3	3745	1248.33	45.375
590	S	PS	3	3531	1177	0	0	0	3	3531	1177	43.676
591	U	PS	5	3396	679.2	2	207.46	103.73	3	3188.54	1062.85	9.824
592	U	PS	2	744	372	1	136	136	1	608	608	3.642
593	S	PS	3	8283	2761	0	0	0	3	8283	2761	44.247
594	S	9CS	6	10393.08	1732.18	3	1485.08	495.03	3	8908	2969.33	16.219
595	U	PS	2	3771.2	1885.6	1	623.2	623.2	1	3148	3148	2.495
596	U	PS	1	768	768	0	0	0	1	768	768	2.165
597	U	PS	3	1474	491.33	1	65	65	2	1409	704.5	4.42
598	U	9CS	1	1600	1600	0	0	0	1	1600	1600	2.425
599	S	PS	1	1830	1830	0	0	0	1	1830	1830	31.86
600	S	PS	4	6878	1719.5	0	0	0	4	6878	1719.5	54.51
601	U	PS	2	1938	969	0	0	0	2	1938	969	2.018
602	U	PS	5	4323.73	864.75	3	446.44	148.81	2	3877.29	1938.64	6.06
603	U	9CS	2	1467	733.5	0	0	0	2	1467	733.5	2.707
604	S	9CS	7	14426	2060.86	1	1008	1008	6	13418	2236.33	35.976
605	S	PS	3	4289	1429.67	0	0	0	3	4289	1429.67	55.166
606	S	PS	2	3599	1799.5	0	0	0	2	3599	1799.5	20.473
607	U	PS	1	838.5	838.5	0	0	0	1	838.5	838.5	9.012
608	U	PS	1	1160	1160	0	0	0	1	1160	1160	0.751
609	S	9CS	3	8166	2722	0	0	0	3	8166	2722	28.565
610	U	PS	2	699	349.5	1	91	91	1	608	608	4.28
611	U	PS	2	1870	935	1	120	120	1	1750	1750	6.871
612	S	PS	1	312	312	0	0	0	1	312	312	30.342
613	S	9CS	2	3990	1995	0	0	0	2	3990	1995	20.898
614	S	PS	5	6647	1329.4	1	416	416	4	6231	1557.75	25.399
615	S	PS	5	8108.9	1621.78	1	576	576	4	7532.9	1883.22	33.694
616	U	PS	2	2320	1160	0	0	0	2	2320	1160	6.824
617	U	PS	1	2800	2800	0	0	0	1	2800	2800	7.896
618	U	PS	3	1084.08	361.36	1	94.08	94.08	2	990	495	8.394
619	S	9CS	1	590	590	0	0	0	1	590	590	34.576
620	S	PS	3	6232	2077.33	1	888	888	2	5344	2672	20.089
621	S	12CS	3	7163	2387.67	0	0	0	3	7163	2387.67	44.616
622	U	PS	3	1830	610	2	390	195	1	1440	1440	5.565
623	S	9CS	2	8383	4191.5	0	0	0	2	8383	4191.5	33.041
624	U	PS	1	2516	2516	0	0	0	1	2516	2516	12.625
625	S	PS	5	20829	4165.8	2	1548	774	3	19281	6427	24.119
626	S	PS	2	3027	1513.5	1	46	46	1	2981	2981	25.115
627	S	9CS	5	8442	1688.4	0	0	0	5	8442	1688.4	44.255
628	S	PS	3	3224	1074.67	1	102	102	2	3122	1561	58.286
629	U	PS	1	1800	1800	0	0	0	1	1800	1800	3.083
630	S	9CS	1	9870	9870	0	0	0	1	9870	9870	15.975
631	S	9CS	6	9643.56	1607.26	1	195.36	195.36	5	9448.2	1889.64	39.338
632	S	PS	1	4860	4860	0	0	0	1	4860	4860	49.937
633	U	PS	1	2430	2430	0	0	0	1	2430	2430	5.017
634	S	9CS	2	2497	1248.5	0	0	0	2	2497	1248.5	25.052
635	S	9CS	4	7372	1843	1	240	240	3	7132	2377.33	37.17
636	S	PS	3	5712	1904	1	204	204	2	5508	2754	16.17
637	U	PS	1	2600	2600	0	0	0	1	2600	2600	11.986
638	U	PS	2	2722	1361	1	552	552	1	2170	2170	6.274
639	S	9CS	3	9215	3071.67	0	0	0	3	9215	3071.67	35.726

Samples	Location Type	School Catergory	TT	TA	Average TA	IT	IA	Average IA	OT	OA	Average OA	Location
640	U	PS	2	1384.8	692.4	1	79.8	79.8	1	1305	1305	5.554
641	S	PS	1	1749	1749	0	0	0	1	1749	1749	26.001
642	S	PS	3	5146.4	1715.47	1	363	363	2	4783.4	2391.7	20.731
643	S	PS	4	5147	1286.75	1	608	608	3	4539	1513	43.976
644	S	PS	3	6643	2214.33	1	1000	1000	2	5643	2821.5	22.075
645	S	9CS	3	4642	1547.33	0	0	0	3	4642	1547.33	40.471
646	S	PS	4	5484.8	1371.2	1	370.8	370.8	3	5114	1704.67	37.481
647	U	PS	1	2250	2250	0	0	0	1	2250	2250	8.004
648	S	PS	3	8847	2949	0	0	0	3	8847	2949	19.086
649	S	PS	4	2641	660.25	2	301	150.5	2	2340	1170	53.095
650	U	PS	2	1494.7	747.35	1	161.04	161.04	1	1333.66	1333.66	6.132
651	U	PS	2	1416.84	708.42	1	105.84	105.84	1	1311	1311	3.799
652	U	PS	4	2742.6	685.65	2	907.6	453.8	2	1835	917.5	5.608
653	S	PS	1	2176	2176	0	0	0	1	2176	2176	57.559
654	S	PS	1	5830	5830	0	0	0	1	5830	5830	24.036
655	S	PS	1	421.2	421.2	0	0	0	1	421.2	421.2	23.982
656	S	PS	5	6381.42	1276.28	1	706.42	706.42	4	5675	1418.75	20.837
657	U	PS	3	1070.16	356.72	2	435.1	217.55	1	635.06	635.06	2.309
658	U	PS	3	2608.28	869.43	1	176.28	176.28	2	2432	1216	3.305
659	U	PS	1	2700	2700	0	0	0	1	2700	2700	12.728
660	S	PS	3	858	286	0	0	0	3	858	286	39.474
661	S	9CS	4	19690	4922.5	1	936	936	3	18754	6251.33	35.839
662	S	9CS	7	29128	4161.14	3	1800	600	4	27328	6832	24.268
663	S	PS	1	7200	7200	0	0	0	1	7200	7200	35.102
664	S	PS	1	1023	1023	0	0	0	1	1023	1023	35.67
665	S	PS	2	643	321.5	1	35	35	1	608	608	41.174
666	U	PS	3	1804.91	601.64	1	151.2	151.2	2	1653.71	826.86	9.355
667	S	PS	1	2759.64	2759.64	0	0	0	1	2759.64	2759.64	57.883
668	S	PS	5	8708	1741.6	1	748	748	4	7960	1990	40.566
669	S	PS	5	13602	2720.4	1	249	249	4	13353	3338.25	59.152
670	S	PS	2	4028	2014	0	0	0	2	4028	2014	28.169
671	S	PS	1	1500	1500	0	0	0	1	1500	1500	28.244
672	S	PS	4	4528.03	1132.01	1	763.03	763.03	3	3765	1255	17.572
673	U	PS	3	4189.76	1396.59	1	490.44	490.44	2	3699.32	1849.66	8.241
674	S	9CS	5	6339.56	1267.91	3	654.08	218.03	2	5685.48	2842.74	18.417
675	S	PS	4	4384	1096	1	960	960	3	3424	1141.33	21.454
676	S	PS	4	8397	2099.25	1	540	540	3	7857	2619	27.859
677	U	PS	1	2001	2001	0	0	0	1	2001	2001	8.968
678	U	PS	2	3864	1932	1	608	608	1	3256	3256	13.411
679	U	PS	3	5300.6	1766.87	2	860.6	430.3	1	4440	4440	10.408
680	S	PS	1	3000	3000	0	0	0	1	3000	3000	24.363
681	U	PS	2	2803	1401.5	1	203	203	1	2600	2600	9.588
682	S	PS	2	5979	2989.5	0	0	0	2	5979	2989.5	33.92
683	S	PS	4	6716	1679	1	575	575	3	6141	2047	57.599
684	S	PS	5	6522.68	1304.54	2	756	378	3	5766.68	1922.23	16.379
685	S	PS	3	4700	1566.67	1	420	420	2	4280	2140	15.819
686	S	9CS	3	3964	1321.33	0	0	0	3	3964	1321.33	44.296
687	S	PS	3	7137	2379	0	0	0	3	7137	2379	35.431
688	U	PS	1	2575.06	2575.06	0	0	0	1	2575.06	2575.06	10.758
689	U	PS	2	2100	1050	1	100	100	1	2000	2000	8.408
690	U	PS	1	2552	2552	0	0	0	1	2552	2552	10.26
691	S	PS	3	6242	2080.67	0	0	0	3	6242	2080.67	15.099
692	U	PS	2	4040	2020	1	360	360	1	3680	3680	9.427
693	U	PS	3	3953	1317.67	1	345	345	2	3608	1804	11.709
694	U	12CS	3	7419	2473	0	0	0	3	7419	2473	11.448
695	U	PS	3	6334.2	2111.4	1	678.2	678.2	2	5656	2828	11.674
696	S	PS	4	8541.7	2135.43	1	732.7	732.7	3	7809	2603	55.683
697	S	PS	4	13562	3390.5	2	796	398	2	12766	6383	18.446
698	S	PS	2	2271	1135.5	0	0	0	2	2271	1135.5	19.362
699	S	9CS	2	8080	4040	0	0	0	2	8080	4040	35.24
700	U	PS	4	2702.05	675.51	2	241.65	120.83	2	2460.4	1230.2	3.903
701	U	PS	3	6546	2182	1	608	608	2	5938	2969	11.013
702	U	PS	1	4407	4407	0	0	0	1	4407	4407	9.434
703	S	9CS	6	13284.4	2214.07	1	1247.4	1247.4	5	12037	2407.4	36.464
704	U	PS	4	7090	1772.5	1	864	864	3	6226	2075.33	10.003
705	U	PS	1	3500	3500	0	0	0	1	3500	3500	11.184
706	S	9CS	4	5974	1493.5	1	640	640	3	5334	1778	26.851
707	S	PS	4	7176	1794	1	338	338	3	6838	2279.33	35.933
708	S	9CS	3	8285	2761.67	0	0	0	3	8285	2761.67	45.051
709	U	PS	2	5108	2554	0	0	0	2	5108	2554	6.858
710	U	PS	5	10627	2125.4	1	693	693	4	9934	2483.5	9.105

Samples	Location Type	School Catergory	TT	TA	Average TA	IT	IA	Average IA	OT	OA	Average OA	Location
711	S	PS	1	4320	4320	0	0	0	1	4320	4320	34.49
712	U	PS	1	1564	1564	0	0	0	1	1564	1564	10.915
713	U	12CS	3	7708	2569.33	1	1340	1340	2	6368	3184	12.181
714	U	PS	3	4298.75	1432.92	1	360	360	2	3938.75	1969.38	9.043
715	U	12CS	3	18656	6218.67	1	1800	1800	2	16856	8428	14.9
716	U	PS	1	4080	4080	0	0	0	1	4080	4080	7.724
717	U	PS	2	2371	1185.5	0	0	0	2	2371	1185.5	13.033
718	U	PS	3	3911.6	1303.87	1	201.6	201.6	2	3710	1855	11.222
719	S	9CS	2	7376	3688	0	0	0	2	7376	3688	22.116
720	S	PS	5	4289	857.8	1	44	44	4	4245	1061.25	55.974
721	S	PS	1	880	880	0	0	0	1	880	880	37.158
722	U	PS	2	2914.87	1457.43	0	0	0	2	2914.87	1457.43	8.935
723	S	PS	2	2708	1354	0	0	0	2	2708	1354	21.335
724	S	PS	1	4032	4032	0	0	0	1	4032	4032	16.755
725	S	9CS	5	1717.58	343.52	3	1631.68	543.89	2	85.9	42.95	35.328
726	S	PS	2	4364	2182	0	0	0	2	4364	2182	35.241
727	U	9CS	3	5184.3	1728.1	1	696.3	696.3	2	4488	2244	9.911
728	S	PS	2	4746	2373	0	0	0	2	4746	2373	35.279
729	U	PS	6	12262.1	2043.68	3	574.1	191.37	3	11688	3896	11.03
730	S	12CS	4	8220	2055	1	1344	1344	3	6876	2292	18.999
731	S	9CS	6	14552	2425.33	2	1256	628	4	13296	3324	17.327
732	S	PS	3	4459	1486.33	0	0	0	3	4459	1486.33	35.895
733	S	9CS	5	8349	1669.8	0	0	0	5	8349	1669.8	28.499
734	S	PS	2	5700	2850	0	0	0	2	5700	2850	43.672
735	U	PS	2	1376	688	0	0	0	2	1376	688	9.815
736	S	PS	5	5300	1060	0	0	0	5	5300	1060	25.854
737	S	12CS	2	1830	915	1	1530	1530	1	300	300	21.8
738	S	PS	1	520	520	0	0	0	1	520	520	34.471
739	S	PS	1	5336	5336	0	0	0	1	5336	5336	26.389
740	S	PS	3	6232	2077.33	1	391	391	2	5841	2920.5	25.422
741	S	9CS	4	6830	1707.5	1	864	864	3	5966	1988.67	41.91
742	S	PS	2	23674	11837	0	0	0	2	23674	11837	22.964
743	S	9CS	4	8715	2178.75	1	250	250	3	8465	2821.67	30.885
744	S	PS	4	12513	3128.25	1	1080	1080	3	11433	3811	19.917
745	S	12CS	9	27340	3037.78	2	3598	1799	7	23742	3391.71	15.514
746	U	9CS	1	805	805	0	0	0	1	805	805	2.256
747	U	PS	1	2695	2695	0	0	0	1	2695	2695	9.267
748	U	PS	3	2877	959	0	0	0	3	2877	959	11.647
749	U	PS	1	2480	2480	0	0	0	1	2480	2480	9.264
750	S	PS	4	811	202.75	0	0	0	4	811	202.75	55.817
751	U	9CS	2	3182.39	1591.19	0	0	0	2	3182.39	1591.19	4.129
752	U	PS	2	4316	2158	1	714	714	1	3602	3602	6.084
753	U	PS	3	2574	858	1	259	259	2	2315	1157.5	12.235
754	U	PS	2	2149	1074.5	0	0	0	2	2149	1074.5	1.697
755	U	PS	1	2201.6	2201.6	0	0	0	1	2201.6	2201.6	10.125
756	U	PS	4	4191.47	1047.87	0	0	0	4	4191.47	1047.87	9.82
757	U	PS	2	5450	2725	1	1050	1050	1	4400	4400	13.936
758	S	PS	5	8670	1734	0	0	0	5	8670	1734	40.238
759	U	PS	1	1740	1740	0	0	0	1	1740	1740	2.607
760	S	PS	1	1216	1216	0	0	0	1	1216	1216	42.253
761	S	PS	1	5000	5000	0	0	0	1	5000	5000	25.41
762	S	PS	2	4416	2208	0	0	0	2	4416	2208	32.732
763	U	PS	5	3197	639.4	1	478	478	4	2719	679.75	6.593
764	S	PS	2	5288	2644	0	0	0	2	5288	2644	44.266
765	U	9CS	2	6336	3168	0	0	0	2	6336	3168	6.523
766	U	PS	1	390	390	0	0	0	1	390	390	1.06
767	S	PS	4	10628	2657	1	640	640	3	9988	3329.33	16.853
768	U	PS	1	1596	1596	0	0	0	1	1596	1596	12.605
769	U	PS	1	300	300	0	0	0	1	300	300	0.977
770	U	PS	1	924	924	0	0	0	1	924	924	3.318
771	S	PS	2	4467	2233.5	0	0	0	2	4467	2233.5	26.209
772	S	PS	1	1760	1760	0	0	0	1	1760	1760	15.246
773	S	9CS	3	9724	3241.33	1	1198	1198	2	8526	4263	21.372
774	U	PS	1	1155	1155	0	0	0	1	1155	1155	9.474
775	U	PS	2	808	404	1	200	200	1	608	608	9.251
776	U	PS	6	8490	1415	2	608	304	4	7882	1970.5	12.47
777	S	PS	6	6973	1162.17	0	0	0	6	6973	1162.17	37.558
778	S	PS	9	6434	714.89	2	609	304.5	7	5825	832.14	57.021
779	U	PS	1	299	299	0	0	0	1	299	299	2.306
780	S	PS	2	4776	2388	1	576	576	1	4200	4200	19.953
781	S	PS	4	1841	460.25	0	0	0	4	1841	460.25	34.716

Samples	Location Type	School Catergory	TT	TA	Average TA	IT	IA	Average IA	OT	OA	Average OA	Location
782	U	PS	1	640	640	0	0	0	1	640	640	3.068
783	S	9CS	1	420	420	0	0	0	1	420	420	20.123
784	S	PS	2	3908	1954	0	0	0	2	3908	1954	19.342
785	U	PS	1	875	875	1	875	875	0	0	0	13.584
786	U	PS	5	10260	2052	1	240	240	4	10020	2505	14.537
787	U	PS	3	1932.47	644.16	1	332.22	332.22	2	1600.25	800.12	7.159
788	U	PS	3	5674	1891.33	1	792	792	2	4882	2441	11.242
789	U	PS	4	7814	1953.5	1	756	756	3	7058	2352.67	6.789
790	U	9CS	3	6806.1	2268.7	1	195.3	195.3	2	6610.8	3305.4	2.596
791	U	PS	3	4946.78	1648.93	1	632.78	632.78	2	4314	2157	7.757
792	U	PS	8	10041	1255.12	1	704	704	7	9337	1333.86	9.855
793	S	PS	2	4300	2150	0	0	0	2	4300	2150	13.553
794	U	PS	4	5502.08	1375.52	2	963.08	481.54	2	4539	2269.5	10.744
795	U	12CS	3	2436.48	812.16	2	270.48	135.24	1	2166	2166	5.8
796	S	PS	2	4536	2268	1	336	336	1	4200	4200	33.413
797	S	9CS	1	608	608	0	0	0	1	608	608	41.92
798	U	PS	1	3600	3600	0	0	0	1	3600	3600	10.699
799	U	9CS	4	8714.4	2178.6	1	720	720	3	7994.4	2664.8	7.568
800	U	9CS	5	3626	725.2	1	459	459	4	3167	791.75	11.759
801	S	PS	6	8691.95	1448.66	1	509.95	509.95	5	8182	1636.4	33.384
802	U	PS	3	3018	1006	1	660	660	2	2358	1179	8.223
803	U	PS	2	999	499.5	1	306	306	1	693	693	3.852
804	U	PS	3	3322.7	1107.57	1	559	559	2	2763.7	1381.85	13.504
805	U	PS	2	4501.64	2250.82	0	0	0	2	4501.64	2250.82	6.761
806	S	PS	2	732	366	1	108	108	1	624	624	21.405
807	U	PS	1	912	912	0	0	0	1	912	912	1.137
808	S	PS	6	4195.1	699.18	1	548.1	548.1	5	3647	729.4	22.063
809	S	PS	2	6400	3200	0	0	0	2	6400	3200	48.96
810	S	9CS	2	4434	2217	1	480	480	1	3954	3954	29.288
811	U	PS	1	696	696	0	0	0	1	696	696	13.532
812	U	PS	3	3039.75	1013.25	1	193.75	193.75	2	2846	1423	4.855
813	U	12CS	3	5176	1725.33	1	600	600	2	4576	2288	10.716
814	S	PS	1	900	900	0	0	0	1	900	900	25.445
815	U	9CS	3	5600	1866.67	0	0	0	3	5600	1866.67	9.443
816	U	PS	1	608	608	0	0	0	1	608	608	6.524
817	U	PS	3	4359.86	1453.29	1	360.89	360.89	2	3998.97	1999.48	10.677
818	U	PS	1	2214	2214	0	0	0	1	2214	2214	4.868
819	U	PS	5	5088	1017.6	1	1782	1782	4	3306	826.5	13.431
820	S	9CS	4	3632	908	2	174	87	2	3458	1729	83.102
821	U	PS	1	3690	3690	0	0	0	1	3690	3690	13.551
822	U	PS	2	894	447	0	0	0	2	894	447	12.957
823	U	12CS	1	4440	4440	0	0	0	1	4440	4440	13.249
824	S	PS	1	3710	3710	0	0	0	1	3710	3710	31.943
825	U	PS	2	4904	2452	1	704	704	1	4200	4200	5.778
826	S	PS	2	3865	1932.5	0	0	0	2	3865	1932.5	138.464
827	S	PS	1	608	608	0	0	0	1	608	608	24.679
828	S	PS	2	5218	2609	0	0	0	2	5218	2609	34.839
829	S	PS	4	9669	2417.25	2	1225	612.5	2	8444	4222	24.006
830	U	PS	3	4769	1589.67	1	608	608	2	4161	2080.5	13.248
831	U	PS	2	5819.5	2909.75	1	973.5	973.5	1	4846	4846	7.176
832	S	PS	4	7865	1966.25	1	989	989	3	6876	2292	17.079
833	S	PS	2	4950	2475	0	0	0	2	4950	2475	17.945
834	S	PS	1	4800	4800	0	0	0	1	4800	4800	18.985
835	S	9CS	4	20084.96	5021.24	1	1554	1554	3	18530.96	6176.99	15.831
836	S	PS	2	5666	2833	0	0	0	2	5666	2833	20.207
837	S	PS	1	840	840	0	0	0	1	840	840	23.47
838	S	PS	1	1740	1740	0	0	0	1	1740	1740	34.387
839	S	PS	3	2184	728	1	66	66	2	2118	1059	56.615
840	S	9CS	2	8809	4404.5	0	0	0	2	8809	4404.5	64.205
841	U	PS	7	10545.08	1506.44	2	1220.08	610.04	5	9325	1865	9.404
842	U	PS	1	1750	1750	0	0	0	1	1750	1750	5.716
843	U	PS	3	6176	2058.67	1	800	800	2	5376	2688	8.716
844	U	PS	3	4633.06	1544.35	1	87.12	87.12	2	4545.94	2272.97	11.901
845	S	PS	2	7740	3870	1	608	608	1	7132	7132	35.791
